# Antiretroviral Therapy Uptake, Attrition, Adherence and Outcomes among HIV-Infected Female Sex Workers: A Systematic Review and Meta-Analysis

**DOI:** 10.1371/journal.pone.0105645

**Published:** 2014-09-29

**Authors:** Elisa Mountain, Sharmistha Mishra, Peter Vickerman, Michael Pickles, Charles Gilks, Marie-Claude Boily

**Affiliations:** 1 Department of Infectious Disease Epidemiology, Imperial College London, London, United Kingdom; 2 Division of Infectious Diseases, St. Michael's Hospital, University of Toronto, Toronto, Canada; 3 Li Ka Shing Knowledge Institute, University of Toronto, Toronto, Canada; 4 School of Social and Community Medicine, University of Bristol, Bristol, United Kingdom; 5 School of Population Health, University of Queensland, Brisbane, Australia; University of Pittsburgh, United States of America

## Abstract

**Purpose:**

We aimed to characterize the antiretroviral therapy (ART) cascade among female sex workers (FSWs) globally.

**Methods:**

We systematically searched PubMed, Embase and MEDLINE in March 2014 to identify studies reporting on ART uptake, attrition, adherence, and outcomes (viral suppression or CD4 count improvements) among HIV-infected FSWs globally. When possible, available estimates were pooled using random effects meta-analyses (with heterogeneity assessed using Cochran's Q test and I^2^ statistic).

**Results:**

39 studies, reporting on 21 different FSW study populations in Asia, Africa, North America, South America, and Central America and the Caribbean, were included. Current ART use among HIV-infected FSWs was 38% (95% CI: 29%–48%, I^2^ = 96%, 15 studies), and estimates were similar between high-, and low- and middle-income countries. Ever ART use among HIV-infected FSWs was greater in high-income countries (80%; 95% CI: 48%–94%, I^2^ = 70%, 2 studies) compared to low- and middle-income countries (36%; 95% CI: 7%–81%, I^2^ = 99%, 3 studies). Loss to follow-up after ART initiation was 6% (95% CI: 3%–11%, I^2^ = 0%, 3 studies) and death after ART initiation was 6% (95% CI: 3%–11%, I^2^ = 0%, 3 studies). The fraction adherent to ≥95% of prescribed pills was 76% (95% CI: 68%–83%, I^2^ = 36%, 4 studies), and 57% (95% CI: 46%–68%, I^2^ = 82%, 4 studies) of FSWs on ART were virally suppressed. Median gains in CD4 count after 6 to 36 months on ART, ranged between 103 and 241 cells/mm^3^ (4 studies).

**Conclusions:**

Despite global increases in ART coverage, there is a concerning lack of published data on HIV treatment for FSWs. Available data suggest that FSWs can achieve levels of ART uptake, retention, adherence, and treatment response comparable to that seen among women in the general population, but these data are from only a few research settings. More routine programme data on HIV treatment among FSWs across settings should be collected and disseminated.

## Introduction

By reducing HIV viral load and helping to restore immune function, antiretroviral therapy (ART) has led to substantial reductions in HIV-attributable mortality and morbidity and has greatly improved the quality of life for people living with HIV. Evidence also indicates that individuals on effective ART are less likely to transmit HIV [1]–[5]. This evidence has sparked great interest in ART-based HIV prevention approaches, including ‘Treatment as Prevention’ (TasP), which aims to expand ART coverage among HIV-infected individuals in order to help reduce HIV transmission at a population level [6].

Successful treatment can sustain viral suppression and lead to immunological improvement among those that are HIV-infected, but requires that individuals engage and remain in the HIV care cascade. This cascade involves a series of actions, starting with HIV screening/testing, and followed by linkage to HIV care after HIV diagnosis, retention in pre-ART care prior to ART initiation, initiation of ART once eligible for treatment, retention on treatment once ART is started, and then maintenance of good ART adherence in order to achieve viral suppression and immunological improvement [7]–[9]. However, evidence indicates that many individuals are lost at each stage of the cascade, and many individuals are diagnosed late, only initiating ART at the onset of symptoms [8]–[11].

Female sex workers (FSWs) are a population at high risk of acquiring and transmitting HIV infection, and in many HIV epidemics bear a disproportionately larger burden of HIV [12]–[14]. Worldwide, the HIV prevalence among FSWs is 12%, ranging between 1.7% in the Middle East and North Africa to 36.9% in Sub-Saharan Africa, and FSWs have a pooled odds ratio of HIV infection compared to women in the general population of 14 [13]. Thus, FSWs remain a key population for HIV prevention strategies. Ensuring high levels of ART uptake, adherence and retention among FSWs, would provide not only individual benefits to HIV-infected FSWs, but could also help reduce HIV transmission at the population level [15], [16].

With the high burden of HIV among FSWs and the potential merits of expanding ART in populations at high risk of transmitting HIV [15], [17], it is crucial to understand the extent to which FSWs currently access ART, and continue ART with good adherence. We conducted a systematic review to summarise the existing information on the following events in the HIV care cascade among FSWs globally: ART uptake, ART attrition, adherence, and treatment response (viral suppression and CD4 count improvements).

## Methods

Our systematic review and meta-analysis was conducted and reported in accordance with PRISMA and MOOSE guidelines (see Checklist S1) [18], [19].

### Search Strategy

Our search strategy was conducted in three stages. First, we searched PubMed, Embase and MEDLINE for studies published up until 10^th^ March 2014. The search terms (keywords and medical subject heading terms) reflected three key domains: HIV/AIDS, FSWs and ART (see Table 1 for combinations of search terms used). There were no language restrictions. Following removal of duplicate references, abstracts were screened for exclusion. If studies were not excluded after abstract screening, the full text of the article was retrieved and evaluated for eligibility. Second, we hand-searched the reference lists of all eligible studies and relevant review articles identified in the database search for additional publications. Finally, for completion, whenever an eligible study analysed data from a specific FSW cohort, a supplementary database search was conducted, using the name and location of the FSW cohort and corresponding author names as search terms, to find any additional publications. Authors were contacted for additional information and supplementary data when needed. One reviewer conducted the search, reviewed abstracts, and evaluated full text articles for eligibility. Any queries on article eligibility were discussed and resolved with other team members.

**Table 1:** Search Terms Used For Search Strategy.

Domain	Search Terms
HIV	“HIV” OR “human immunodeficiency virus” OR “AIDS” OR “acquired immune deficiency syndrome”
	**AND**
FSWs	“FSW” OR “FSWs” OR “CSW” OR “CSWs” OR “commercial sex” OR “female sex worker*” OR “commercial sex work*” OR “sex-work*” OR “sexwork*” OR “sex work*” OR “prostitute*” OR “prostitution” OR “transactional sex” OR “paid sex” OR “money for sex” OR “sex for money” OR “paid for sex” OR “sex in exchange for money” OR ((“core group” OR “high risk” OR “high-risk” OR highrisk”) AND (“female*” OR “women” or “woman”))
	**AND**
ART	“ART” OR “antiretroviral*” OR “anti-retroviral*” OR “HAART” OR “highly active antiretroviral therapy” OR “cART” OR “combined antiretroviral therapy”

### Inclusion and Exclusion Criteria

We included observational or intervention studies with a sample size of at least 10 FSWs that reported estimates or sufficient data to derive the following ART cascade outcomes: ART uptake (specifically the fraction of all HIV-infected individuals or the fraction of all ART-eligible individuals, who either initiated ART within a specified follow-up period, currently use ART, or ever used ART (no time-frame for ART initiation specified)), ART attrition (specifically the fraction using ART who were either lost-to-follow-up, died or discontinued ART, or the fraction of treatment-experienced individuals no longer on ART), ART adherence (specifically the fraction achieving a predefined threshold of adherence e.g. ≥90%, ≥95%, 100%), viral suppression (specifically the fraction with undetectable plasma viral load following ART initiation), and CD4 counts at and/or after ART initiation (specifically the median CD4 count, median CD4 count gain, or fraction with CD4 counts <200, 200–499 or >500 cells/mm^3^). We included studies which reported on the same study population if the studies provided estimates of different ART cascade outcomes for that study population or provided estimates for that study population on the same ART cascade outcome over different time periods.

We included studies that enrolled active or former FSWs, women who engage in sex work or transactional sex, or women who exchange sex for money, drugs or gifts. “Active” FSWs were defined as women who either reported sex work as their employment at enrolment, or within the previous 6 months. “Former” FSWs were those that either reported sex work as a former occupation at enrolment, reported transactional sex more than 6 months ago, or were no longer sexually active. We excluded review articles, mathematical modelling studies, qualitative studies and conference/oral/poster abstracts.

### Data Extraction

One reviewer extracted data from included articles, and any queries during data extraction were discussed and resolved with other team members. Variables extracted included study characteristics (e.g. study design, study period and sample size of FSWs), participant characteristics (e.g. HIV status and illicit drug use), outcome estimates (or the data to calculate them), and the time period over which estimates were measured. Exact binomial confidence intervals (CI) for proportions were derived when not provided. Each study could provide information on multiple ART cascade outcomes and/or on the same ART cascade outcome over different time periods. Multiple studies could provide different ART cascade estimates for the same study population.

We also extracted information on ART initiation criteria and ART administration in order to provide a background to the local context for included studies. To assess risk of bias in included studies, we also extracted the following study characteristics: study setting and recruitment, type of sampling, and outcome measurement methods. No studies were excluded from the systematic review or meta-analysis on the basis of this assessment, but the potential impact of these characteristics on study results was considered when interpreting overall findings.

### Data Analysis

We firstly describe key characteristics of all included studies and the total number that provide estimates for the different ART cascade outcomes. We then summarise the results from different studies for each outcome. Given that several studies could provide information on the same study population, we give both the number of studies (N_s_) and the number of independent study populations (N_p_) when summarising the study characteristics and ART cascade outcome estimates.

All available study estimates are presented on forest plots in the main text and are also reported in Tables S1–S5. On all forest plots, study estimates are grouped by country income (high-income (HI) versus low- and middle-income (LMI)), and ordered by study-period, follow-up period, or time on ART.

For each outcome with at least 2 study estimates available from different study populations, we calculated an overall pooled estimate, and when possible performed sub-group meta-analyses by country income (i.e. HI versus LMI). These results are reported in the main text and are also presented on forest plots. As time period of data collection could influence HIV treatment outcomes, in particular ART uptake outcomes, we did additional sub-group analyses for ART uptake outcomes in order to address this issue. If the time period of data collection for any estimates of ART uptake spanned the pre-HAART and HAART era, sub-group meta-analyses were performed if possible by year of data collection (i.e. data collected in the pre-HAART to HAART era versus data collected in the HAART era) for that ART uptake outcome. These results are reported in the main text. We did not pool study estimates for outcomes relating to median CD4 count. For all other outcomes, we pooled study estimates using a DerSimonian and Laird random effects model on the logit scale, and then back-transformed the overall pooled estimate to the original scale. We assessed heterogeneity across study-estimates using the Cochran Q homogeneity test, which assesses whether differences in study estimates are due to chance alone (typically a p-value <0.1 or <0.05 indicates heterogeneity between study estimates), and the I^2^ statistic, which measures the percentage of variation across study estimates that is due to heterogeneity rather than chance (the higher the I^2^ value the greater the heterogeneity between study estimates) [20], [21]. Meta-analyses were conducted using the ‘meta’ package in R (Version 3.0.0).

When pooling estimates, we only included study estimates with sample sizes of at least 10 FSWs. Study estimates with an unknown numerator and denominator were not included in any pooled estimates, and for ART uptake outcomes any study estimates with an unknown study period were not included in pooled estimates. If a study provided estimates of an outcome over different time-periods, we only used one estimate (the most recent estimate from each study) for pooling. If more than one study reported estimates of a given outcome for the same study population, we only used one estimate per study population (the study estimate with the largest sample size) for pooling. Study estimates that were included in overall or subgroup pooled estimates are indicated in forest plots and tables with a star symbol (*).

## Results

### Search Results and Study Characteristics

**Figure 1:**
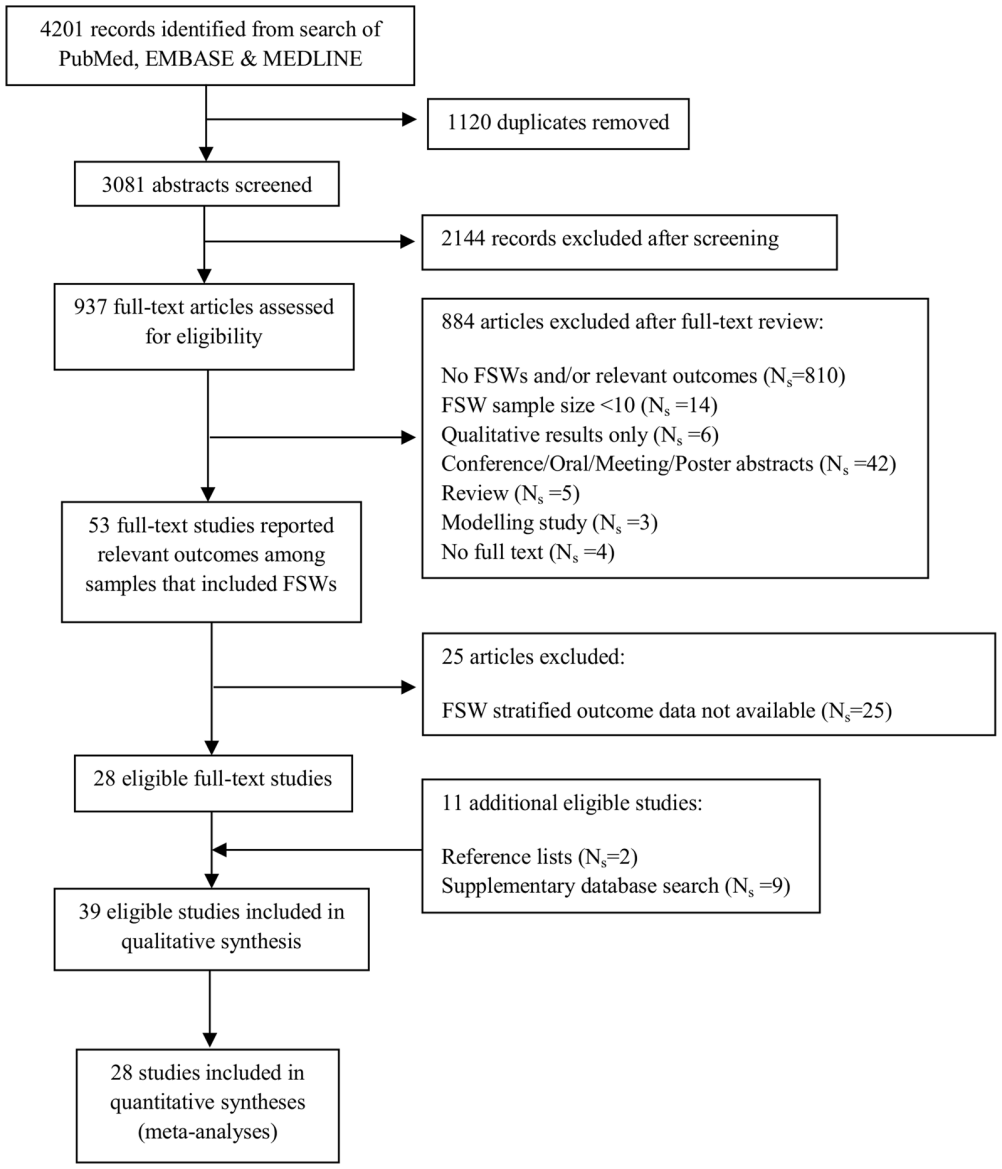
Flow diagram of search strategy and study selection process. FSW  =  female sex worker, ART  =  antiretroviral therapy, N_s_ =  number of studies.

**Figure 2:**
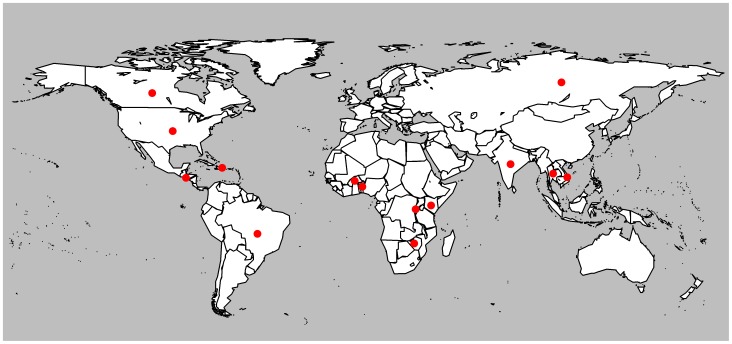
Map of Study Locations. Red dots highlight the countries where included studies were conducted.

The search strategy and process of article selection is described in Figure 1. Of 3081 unique publications identified in our original search, 28 met the inclusion criteria (Figure 1). Additional studies were then identified from the supplementary database search (N_s_ = 9) and reference lists (N_s_ = 2) (Figure 1). In total, 39 studies (N_s_ = 39) identified in the search were eligible to be included in our review (Figure 1), providing data on 21 independent FSW study populations (N_p_ = 21) and at least 4,700 HIV-infected FSWs [22]–[60]. Table 2 summarises the key characteristics of the 39 included studies and their reported outcomes. Figure 2 shows the geographical location of the study settings. Study characteristics that were extracted to help assess risk of bias are shown in Table S6.

**Table 2:** Study Characteristics and Outcomes Reported.

Study Population	Country/Population Code	Study [Reference]	Study Design	Study Period	FSW Sample Size	FSW Characteristics	ART Cascade Outcomes				
							Uptake	Attrition	Adherence	VS	CD4
Abriendo Puertas Intervention Project	Dominican Republic 1	Donastorg *et al*, 2014 [53] a	CS	2012–2013	268	Active, HIV +ve, Drug use (24%)	✓	✓	✓	✓	
ACCESS cohort	Canada 1	Reddon *et al*, 2011 [45] c	PC	2005–2011	88	Active, HIV +ve, IDU	✓				
Canadian Co-infection Cohort	Canada 2	Cox *et al*, 2014 [58] a	PC	2003–2013	43	Active, HIV +ve, IDU or crack	✓				
Chiang Rai Health Club FSW Cohort	Thailand 1	Kilmarx *et al*, 2000 [22]	PC	1991–1998	194	Active, HIV +ve	✓				
El Salvador FSW and MSM Study	El Salvador 1	Dennis *et al*, 2013 [52]	CS	2008	848	Active, HIV +ve (5%)	✓				
FSW and GP Comparative Study	Benin 1	Diabaté *et al*, 2011 [44]	PC	2008–2010	53	Active, HIV +ve, On ART					✓
FSW Focus Group Study	India 1	Chakrapani *et al*, 2009 [46] a	CS	2007	19	Active, HIV +ve	✓				
HERMITAGE RCT Cohort	Russia 1	Tyurina *et al*, 2013 [59] a	PC	2007–2011	42	Active, HIV +ve, Risky drinkers	✓				
Maka Project Partnership/WISH Drop-In Centre Society	Canada 3	Deering *et al*, 2009 [23] a	PDI	2007–2008	20	Active + Former, HIV +ve, IDU (40%)			✓		
		Shannon *et al*, 2005 [24]	CS	2003	159	Active + Former, HIV +ve (21%), IDU (57%)	✓	✓			
Karnataka Multi-District FSW Study	India 2	Jadhav *et al*, 2013 [55]	CS	2011	603	Active, HIV +ve	✓				
Mombasa FSW cohort	Kenya 1	Balkus *et al*, 2013 [49]	PC	1993–2010	282	Active, HIV +ve, T.*vaginalis* infection	✓				
		Day et al, 2013 [51]	PC	2004–2010	159	Active, HIV +ve, On ART		✓			✓
		Graham *et al*, 2013 [54]	PC	1993–2009	306	Active, HIV +ve	✓				
		Graham *et al*, 2012 [29] b	PC	2005–2009	102	Active + Former, HIV +ve, On ART		✓			✓
		Graham *et al*, 2011 [26]	PC	2004–2008	36	Active, HIV +ve, On ART, Genital ulcers					✓
		Masese *et al*, 2011[25]	PC	2004–2008	41	Active, HIV +ve, On ART, T.*vaginalis* infection		✓	✓		✓
		McClelland *et al*, 2011 [33] c	CS	1993–2006	571	Active, HIV +ve	✓				
		Gitau *et al*, 2010 [31]	PC	2004–2008	30	Active, HIV +ve, On ART, Cervicitis infection					✓
		Graham *et al*, 2010 [28] b	PC	2005–2008	102	Active + Former, HIV +ve, On ART		✓		✓	✓
		McClelland *et al*, 2010 [32] a	PC	1993–2008	966	Active, HIV +ve	✓				✓
		Graham *et al*, 2009 [27]	PC	2004–2008	134	Active, HIV +ve, On ART					✓
		Graham *et al*, 2007 [30] a	PC	2004	21	Active, HIV +ve, On ART			✓		✓
Multicentre Vietnam Study	Vietnam 1	Dean *et al*, 2011 [43] a	CS	2008–2009	147	Active, HIV +ve	✓			✓	
Multicity Study of YPLH	USA 1	Comulada *et al*, 2003 [48] a	CS	1999–2000	21	Active + Former, HIV +ve	✓	✓	✓		
Multisite Zimbabwe FSW Study	Zimbabwe 1	Cowan *et al*, 2013 [50]	CS	2009	836	Active, HIV +ve (57%)	✓				
Nairobi FSW cohort	Kenya 2	Mawji *et al*, 2012 [35] a	RCC	2001–2006	62	Active, HIV +ve, On ART					✓
		McKinnon *et al*, 2010 [34] a	PC	1986–2009	607	Active, HIV +ve	✓				✓
		Lester *et al*, 2009 [36]	CS	Not reported	57	Active, HIV +ve	✓				
North Mombasa Clinic Cohort	Kenya 3	Graham *et al*, 2013 [60] a	PC	2005–2011	108	Active, HIV +ve	✓	✓	✓		
*Payana* FSW Cohort	India 3	Becker *et al*, 2012 [40] a	PC	2008–2009	45	Active, HIV +ve, Died during follow-up	✓				
Port of Imbutiba Sex Worker Study	Brazil 1	Schuelter-Trevisol *et al*, 2007 [47]	CS	2003–2004	90	Active, HIV +ve (4%), Illicit Drug Users (52%)	✓				
Project HOPE	USA 2	Kalokhe *et al*, 2012 [41] c	CS	2006–2010	46	Active, HIV +ve, Crack cocaine use	✓				
Project Ubuzima FSW HIV incidence study	Rwanda 1	Braunstein *et al*, 2011 [42]	PC	2006–2008	141	Active + Former, HIV +ve	✓		✓		✓
Yerelon FSW Cohort	Burkina Faso 1	Low et al, 2014 [56]	PC	2004–2011	199	Active, HIV +ve, On ART				✓	✓
		Low et al, 2014 [57]	PC	2007–2011	258	Active, HIV +ve	✓				
		Huet *et al*, 2011 [39]	PC	2003–2007	47	Active, HIV +ve, On ART		✓	✓	✓	✓
		Konate *et al*, 2011 [38]	PDI	2003–2005	658	Active, HIV +ve	✓	✓	✓	✓	✓
		Low *et al*, 2011 [37]	PC	2003–2006	767	Active, HIV +ve	✓				✓

FSW – female sex worker, ART – antiretroviral therapy, HIV – human immunodeficiency virus, VS – viral suppression, MSM – men who have sex with men, GP – general population, IDU – injecting drug user, PC – prospective cohort, CS – cross-sectional, RCT – randomised controlled trial, RCC – retrospective case-control, PDI – peer driven intervention, YPLH – young people living with HIV, HERMITAGE – HIV's Evolution in Russia – Mitigating Infection Transmission and Alcoholism in a Growing Epidemic, WISH – Women's Information and Safe House, HOPE – Hospital visit is an Opportunity for Prevention and Engagement, ACCESS – AIDS Care Cohort to evaluate Exposure to Survival Services.

a Additional data or clarifications were provided by corresponding author/s.

b Corresponding author confirmed that participants were FSWs from the Mombasa FSW cohort.

c Outcome data is based on entire cohort rather than subset of participants in referenced study; in some cases this data was provided by study authors.

The majority of the 39 included studies were prospective cohort (N_s_ = 24, N_p_ = 11) or cross-sectional (N_s_ = N_p_ = 12) studies. There were also a small number of intervention (N_s_ = N_p_ = 2) and retrospective case-control (N_s_ = N_p_ = 1) studies. Twenty-four studies were conducted in five African countries: Benin (N_s_ = N_p_ = 1), Burkina Faso (N_s_ = 5, N_p_ = 1), Kenya (N_s_ = 16, N_p_ = 3), Rwanda (N_s_ = N_p_ = 1), and Zimbabwe (N_s_ =  N_p_ = 1). Six studies were conducted in two North American countries: Canada (N_s_ = 4, N_p_ = 3) and United States (N_s_ = N_p_ = 2). Six studies were conducted in four Asian countries: India (N_s_ = N_p_ = 3), Russia (N_s_ = N_p_ = 1), Thailand (N_s_ = N_p_ = 1), and Vietnam (N_s_ = N_p_ = 1). Two studies were conducted in two Central America and Caribbean countries: Dominican Republic (N_s_ = N_p_ = 1), and El Salvador (N_s_ = N_p_ = 1), and one study was conducted in South America: Brazil (N_s_ = N_p_ = 1). Twenty-four studies (62%) had fewer than 150 HIV-infected FSW participants, and four studies enrolled HIV-infected FSWs who were also injecting drug users (IDUs) (Table 2).

Of the 39 eligible studies included, 26 reported ART uptake outcomes, 10 reported ART attrition outcomes, 9 reported on adherence to ART, 6 reported on viral suppression, and 17 reported on CD4 counts at and/or after ART initiation (Table 2 and Tables S1–S5). Criteria for ART initiation were reported by only 16 studies, the majority of which reported a CD4 count threshold for ART initiation of 200 cells/mm^3^ (N_s_ = 11) [28]–[30], [32], [37]–[39], [44], [46], [51], [54]. Other CD4 count thresholds used for ART initiation were CD4 count <250 cells/mm^3^ (N_s_ = 2), CD4 count <350 cells/mm^3^ (N_s_ = 1), and any CD4 count (N_s_ = 1) [23], [42], [48], [60], and in one other study the ART initiation criteria changed from CD4 count <200 cells/mm^3^ to CD4 count <350 cells/mm^3^ during the study period (N_s_ = 1) [56]. Other details on ART administration were provided in some studies. For example, studies in Rwanda and India report that ART has been provided free of charge to those with HIV through government ART programmes and ART centres, since 2003 and 2004, respectively [40], [42], [46]. Studies in Canada also report that ART is provided free of charge through provincial drug treatment programmes, and in the United States, free ART is provided to all those living with HIV [23], [45], [48]. In 2005, an ART programme was initiated in the Nairobi FSW cohort in Kenya, as part of the U.S. Presidents Emergency Plan for AIDS Relief (PEPFAR), and FSWs received ART through the research clinic [34]. From 2004, FSWs enrolled in the Mombasa FSW cohort in Kenya received ART through either the study research clinic or other clinics in the area, and FSWs enrolled in the Yérélon FSW cohort in Burkina Faso received ART through either the study research clinic or the Bobo-Dioulasso University Teaching Hospital [29], [32], [33], [37]–[39], [49], [51], [54], [56], [57]. In another study in Mombasa, FSWs received ART at the study research clinic, whilst in Benin FSWs accessed ART at a medical center dedicated to FSWs [44], [60].

### ART Uptake

**Figure 3:**
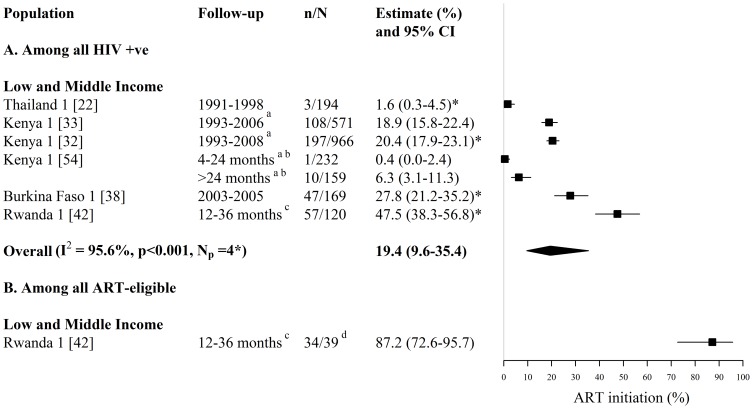
Forest plot of ART initiation among (A) all HIV-infected FSWs and (B) all ART-eligible FSWs. Study estimates are grouped by country income and ordered by follow-up period. The star symbol (*) highlights the individual study estimates (one per study population) included in the pooled overall estimate. I^2^ and p-values are the measures of heterogeneity used. ^a^ ART was provided to FSWs in the Kenyan cohort from 2004, ^b^ Follow-up refers to months since HIV-diagnosis, study recruitment occurred between 1993 and 2009, ^c^ Follow-up refers to months since HIV-diagnosis, study recruitment occurred between 2006 and 2008. ^d^ N refers to HIV-infected ART-eligible FSWs who had enrolled in HIV care following HIV diagnosis. ART  =  antiretroviral therapy, FSW  =  female sex workers, CI  =  confidence interval, n =  number of FSWs with each outcome, N =  sample size of FSWs available for each outcome, N_p_ =  number of independent study populations.

**Figure 4:**
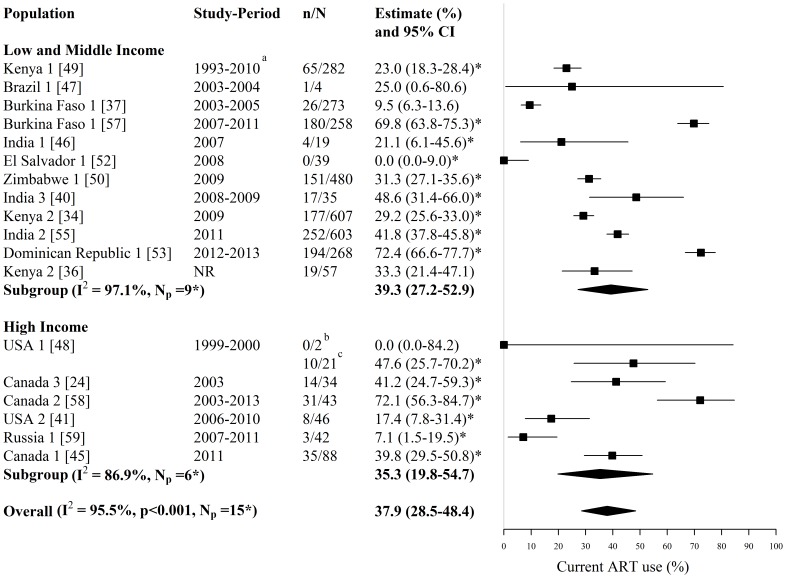
Forest plot of current ART use among HIV-infected FSWs. Study estimates are grouped by country income and ordered by study-period. The star symbol (*) highlights the study estimates (one per study population) included in the pooled overall or subgroup estimates. Only study estimates with a known time period of data collection which were measured among at least 10 FSWs were used for pooling. I^2^ and p-values are the measures of heterogeneity used. ^a^ ART was provided to FSWs in the Kenyan cohort from 2004, ^b^ Sample is ‘active’ FSWs, ^c^ Sample is ‘active’ and ‘former’ FSWs. ART  =  antiretroviral therapy, FSW  =  female sex workers, CI  =  confidence interval, NR  =  not reported, n =  number of FSWs with each outcome, N =  sample size of FSWs available for each outcome, N_p_ =  number of independent study populations.

**Figure 5:**
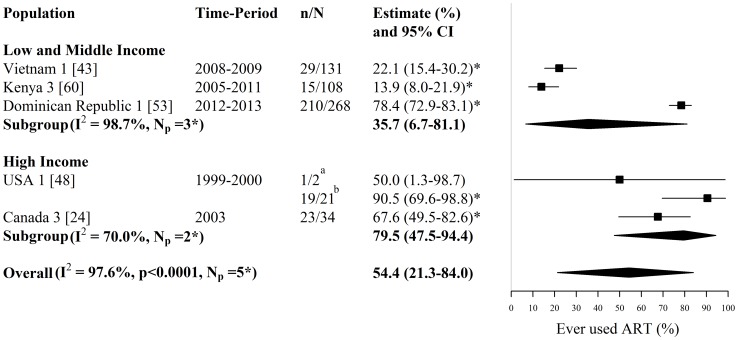
Forest plot of ever ART use among HIV-infected FSWs. Study estimates are grouped by country income and ordered by time-period. The star symbol (*) highlights the study estimates (one per study population) included in the pooled overall or subgroup estimates. I^2^ and p-values are the measures of heterogeneity used. ^a^ Sample is ‘active’ FSWs, ^b^ Sample is ‘active’ and ‘former’ FSWs. ART  =  antiretroviral therapy, FSW  =  female sex workers, CI  =  confidence interval, n =  number of FSWs with each outcome, N =  sample size of FSWs available for each outcome, N_p_ =  number of independent study populations.

Estimates of ART uptake (either ART initiation within specified follow-up periods, current ART use, or ever use of ART (no time-frame for ART initiation specified)), among ART-eligible or all HIV-infected FSWs, were available from 26 studies reporting on 20 independent study populations in Asia, Africa, North America, South America, and Central America and the Caribbean [22], [24], [32]–[34], [36]–[38], [40]–[43], [45]–[50], [52]–[55], [57]–[60] (Figures 3, 4, 5, Table S1). CD4 count criteria for ART initiation were reported by only 8 of these studies (CD4 count ≤200 cells/mm^3^ (N_s_ = 5), CD4 count ≤250 cells/mm^3^ (N_s_ = 1), CD4 count <350 cells/mm^3^ (N_s_ = 1) and any CD4 count (N_s_ = 1)) (Table S1).

Estimates of ART initiation among all HIV-infected FSWs were provided by 6 studies reporting on 4 independent study populations in Asia and Africa (N_s_ = 6, N_p_ = 4) (Figure 3A, Table S1A). Across these 6 studies, 0.4% to 48% of HIV-infected FSWs initiated ART, during follow-up periods which varied between 4 months to 16 years (N_s_ = 6, N_p_ = 4) (Figure 3A, Table S1A). The overall pooled estimate for ART initiation among HIV-infected FSWs, when pooling only one estimate per study population, was 19% (95%CI: 10%–35%, I^2^ = 96%, N_s_ = N_p_ = 4) (Figure 3A). Pooled estimates were lower for studies which collected data on HIV-infected FSWs between the pre-HAART and HAART era (6%; 95%CI: 0%–51%, I^2^ = 96%, N_s_ = N_p_ = 2) than for studies which collected data on HIV-infected FSWs in the HAART era alone (37%; 95%CI: 20%–58%, I^2^ = 91%, N_s_ = N_p_ = 2).

One study in Rwanda provided estimates of ART initiation among ART-eligible FSWs (Figure 3B, Table S1B). In this study, 87% of ART-eligible (CD4 <350 cells/mm^3^) FSWs enrolled in HIV care reported initiating ART within 12 to 36 months of their HIV diagnosis [42] (Figure 3B, Table S1B).

Estimates of current ART use among all HIV-infected FSWs were provided by 18 studies reporting on 16 independent study populations in Asia, Africa, North America, South America, and Central America and the Caribbean (N_s_ = 18, N_p_ = 16) (Figure 4, Table S1A). Across these 18 studies, the fraction of HIV-infected FSWs currently using ART ranged between 0% and 72% (N_s_ = 18, N_p_ = 16) (Figure 4, Table S1A). The overall pooled estimate for current ART use among HIV-infected FSWs, when pooling only one estimate per study population and pooling only those estimates with a known time period of data collection measured among at least 10 FSWs, was 38% (95% CI: 29%–48%, I^2^ = 96%, N_s_ = N_p_ = 15) (Figure 4). Pooled estimates were similar for LMI countries (39%; 95% CI: 27%–53%, I^2^ = 97%, N_s_ = N_p_ = 9) and HI countries (35%; 95% CI: 20%–55%, I^2^ = 87%, N_s_ = N_p_ = 6) (Figure 4). Removing the one LMI country estimate where data on HIV-infected FSWs was collected between the pre-HAART and HAART era had a marginal impact on the pooled estimate for LMI countries (42%; 95% CI: 29%–57%, I^2^ = 97%, N_s_ = N_p_ = 8).

Estimates of ever ART use (i.e. no time-frame for ART initiation specified) among HIV-infected FSWs were provided by five studies reporting on 5 independent study populations in Asia, Africa, North America, and Central America and the Caribbean. Across these 5 studies, the fraction of HIV-infected FSWs who had ever used ART ranged between 14% and 91% (N_s_ = N_p_ = 5) (Figure 5, Table S1A). The overall pooled estimate of ever ART use among HIV-infected FSWs was 54% (95% CI: 21%–84%, I^2^ = 98%, N_s_ = N_p_ = 5). Pooled estimates were lower for LMI countries (36%; 95% CI: 7%–81%, I^2^ = 99%, N_s_ = N_p_ = 3) than HI countries (80%; 95% CI: 48%–94%, I^2^ = 70%, N_s_ = N_p_ = 2) (Figure 5).

### Treatment Attrition

**Figure 6:**
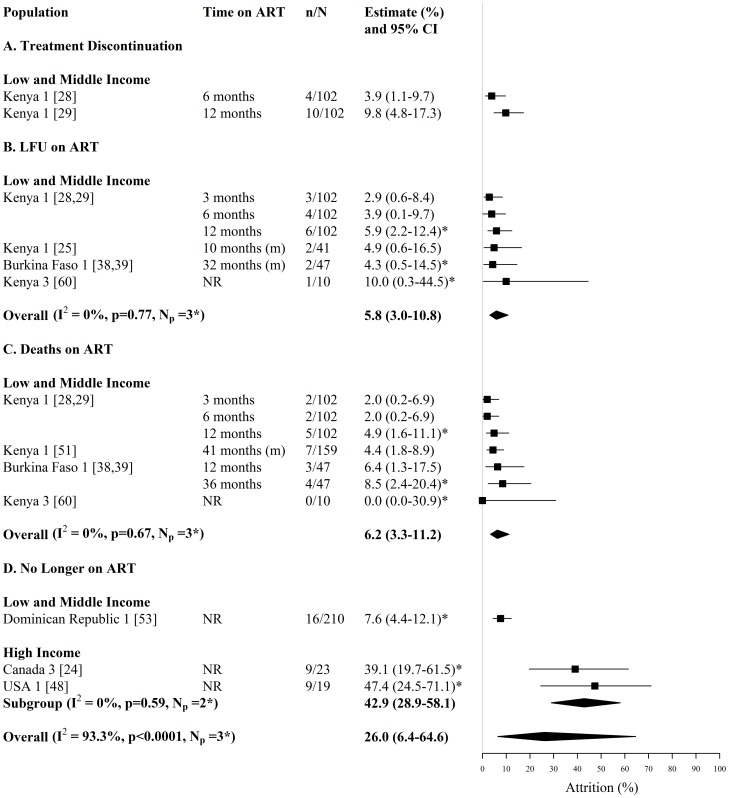
Forest plot of treatment attrition among HIV-infected FSWs, including (A) treatment discontinuation (B) loss to follow-up on ART (C) death on ART and (D) ART-experienced but no longer on ART. Study estimates are grouped by country income and ordered by time on ART. The star symbol (*) highlights the study estimates (one per study population) included in the pooled overall or subgroup estimates. For studies providing estimates over multiple time-periods, only one estimate was used for pooling (the most recent estimate from that study). I^2^ and p-values are the measures of heterogeneity used. ART  =  antiretroviral therapy, FSW  =  female sex workers, CI  =  confidence interval, NR  =  not reported, n =  number of FSWs with each outcome, N =  sample size of FSWs available for each outcome, N_p_ =  number of independent study populations, m =  median, LFU  =  lost to follow-up.

Estimates of treatment attrition (either treatment discontinuation, loss-to-follow-up on ART, death on ART, or ART-experienced but no longer on ART), were available from 10 studies reporting on 6 independent study populations in Africa, North America, and Central America and the Caribbean [24], [25], [28], [29], [38], [39], [48], [51], [53], [60] (Figure 6, Table S2).

Treatment discontinuation was reported by 2 studies reporting on the same study population in Kenya (N_s_ = 2, N_p_ = 1) (Figure 6A, Table S2). In this Kenyan cohort, estimates of treatment discontinuation among FSWs starting ART, increased from 4% at 6 months to 10% at 12 months [28], [29] (Figure 6A, Table S2).

Estimates of loss-to-follow-up among FSWs on ART were provided by 6 studies reporting on 3 independent study populations in Kenya and Burkina Faso (N_s_ = 6, N_p_ = 3) (Figure 6B, Table S2). Across the three different study populations, estimates of loss-to-follow-up after ART initiation were similar, ranging between 3% and 10% after varying times on ART (N_s_ = 6, N_p_ = 3) (Figure 6B, Table S2). In one of the Kenyan cohorts, loss-to-follow-up increased from 3% after 3 months to 6% after 12 months on ART [28], [29], whilst in the Burkina Faso cohort 4% were lost-to-follow-up after 32 months on ART [38], [39]. The overall pooled estimate of loss-to-follow-up on ART, when pooling the most recent study estimate per study population, was 6% (95% CI: 3%–11%, I^2^ = 0%, N_s_ = N_p_ = 3) (Figure 6B).

Estimates of death among FSWs on ART were provided by 6 studies reporting on 3 independent study populations in Kenya and Burkina Faso (N_s_ = 6, N_p_ = 3) (Figure 6C, Table S2). Across the three different study populations, the fraction of FSWs who died after varying times on ART ranged between 0% and 8.5% (N_s_ = 6, N_p_ = 3) (Figure 6C, Table S2). In two of the study populations in Kenya and Burkina Faso the fraction of FSWs who died whilst on ART increased over time [28], [29], [38], [39], [51] (Figure 6C, Table S2). The overall pooled estimate of death on ART, when pooling the most recent study estimate per study population, was 6% (95% CI: 3%–11%, I^2^ = 0%, N_s_ = N_p_ = 3) (Figure 6C). In one of the Kenyan cohorts, total attrition from ART, due to treatment discontinuation, loss-to-follow-up and mortality, was 10% and 21%, after 6 and 12 months on ART, respectively [28], [29].

Estimates of the number of treatment-experienced FSWs that reported no longer receiving ART, were provided by 3 studies reporting on 3 independent study populations in Canada, USA and Dominican Republic (N_s_ = 3, N_p_ = 3) (Figure 6D, Table S2). Across these three studies, the fraction of treatment-experienced FSWs that reported no longer receiving ART ranged between 8% and 47%, with an overall pooled estimate of 26% (95% CI: 6%–65%, I^2^ = 93.3%, N_s_ = N_p_ = 3) (Figure 6D). The pooled estimate for HI countries (43%; 95% CI: 29%–58%, I^2^ = 0%, N_s_ = N_p_ = 2), was higher than the single LMI country estimate (8%, 95% CI: 4%–12%, N_s_ = N_p_ = 1) (Figure 6D, Table S2).

### Adherence

**Figure 7:**
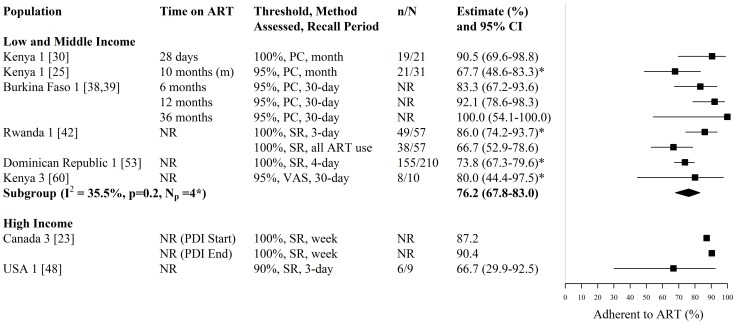
Forest plot of ART adherence among HIV-infected FSWs. Study estimates are grouped by country income and ordered by time on ART. The star symbol (*) highlights the study estimates (one per study population) included in the pooled overall or subgroup estimates. Only study estimates with a known sample size of at least 10 FSWs were used for pooling. For studies providing estimates over multiple time-periods, only one estimate was used for pooling (the most recent estimate from that study). I^2^ and p-values are the measures of heterogeneity used. ART  =  antiretroviral, FSW  =  female sex workers, CI  =  confidence interval, NR  =  not reported, n =  number of FSWs with each outcome, N =  sample size of FSWs available for each outcome, N_p_ =  number of independent study populations, m =  median, PDI  =  peer driven intervention, PC  =  pill count, SR  =  self-report, VAS  =  visual analog scale.

Estimates of adherence (i.e. the fraction achieving a predefined threshold of adherence) were available from 9 studies, reporting on 7 independent study population in Africa, North America, and Central America and the Caribbean [23], [25], [30], [38], [39], [42], [48], [53], [60] (Figure 7, Table S3). Among these 9 studies, three reported the availability of adherence counselling [23], [38], [39], one study reported the use of directly observed therapy [30], and one study reported that monthly support groups were used to promote adherence [60] (Table S3). The fraction of FSWs adherent to treatment was consistently high, ranging from 67% to 100% (N_s_ = 9, N_p_ = 7), across the varying time periods, adherence thresholds (≥90%, ≥95% and 100%), recall periods (past 3 days, past 4 days, past week, past 30 days, past month, and since ART initiation), and methods of assessment (self-report, pill count and visual analog scale) (Figure 7, Table S3). Estimates of the fraction of FSWs 100% adherent to ART were highest shortly after starting directly observed therapy (91% after 28 days on ART [30]) and at the end of a peer-driven intervention (90.4% [23]) (Figure 7, Table S3). Estimates of the fraction of FSWs ≥95% adherent to ART increased from 83% after 6 months on ART to 100% after 36 months on ART in a cohort in Burkina Faso [38], [39] (Figure 7, Table S3). For LMI countries, the pooled estimate of adherence, when pooling only one estimate per study population and pooling only those estimates with a known numerator and denominator, was 76% (95% CI: 68%–83%, I^2^ = 36%, N_s_ = N_p_ = 4) (Figure 7).

### Viral Suppression

**Figure 8:**
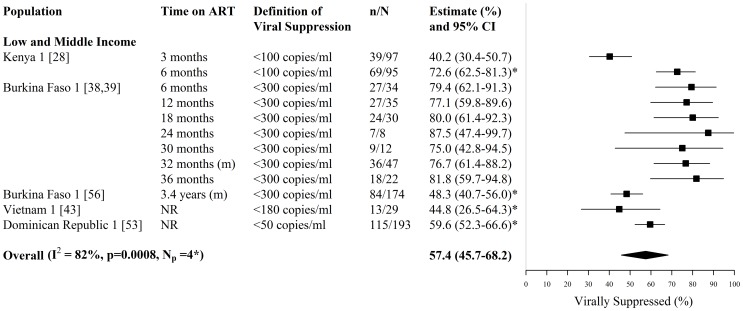
Forest plot of viral suppression among HIV-infected FSWs on ART. Study estimates are grouped by country income and ordered by time on ART. The star symbol (*) highlights the study estimates (one per study population) included in the pooled overall estimate. For studies providing estimates over multiple time-periods, only one estimate was used for pooling (the most recent estimate from that study). I^2^ and p-values are the measures of heterogeneity used. ART  =  antiretroviral, FSW  =  female sex workers, CI  =  confidence interval, NR  =  not reported, n =  number of FSWs with each outcome, N =  sample size of FSWs available for each outcome, N_p_ =  number of independent study populations, m =  median.

Estimates of viral suppression (i.e. the fraction on ART with undetectable plasma viral load), were available from 6 studies reporting on 4 independent study populations in Asia, Africa, and Central America and the Caribbean [28], [38], [39], [43], [53], [56] (Figure 8, Table S4). The fraction of FSWs on ART virally suppressed, ranged between 40%–82% (N_s_ = 6, N_p_ = 4), across varying time periods and definitions of viral suppression (≤50 copies/mm^3^, ≤100 copies/mm^3^, ≤180 copies/mm^3^, ≤300 copies/mm^3^) (Figure 8, Table S4). The lowest study estimate of viral suppression (40%) was reported in the Kenyan study population after 3 months on ART [28], which increased to 73% after 6 months on ART [28] (Figure 8, Table S4). In the Burkina Faso study population, the high fraction of FSWs virally suppressed (79%–88%) after 6 to 36 months on ART, corresponded to similarly high levels of treatment adherence seen over the same time periods for these FSWs (Figure 7 & 8, Tables S3 & S4) [38], [39]. The overall pooled estimate of viral suppression, when pooling one estimate per study population, was 57% (95% CI: 46%–68%, I^2^ = 82%, N_s_ = N_p_ = 4) (Figure 8).

### CD4 Counts at and/or after ART Initiation

**Figure 9:**
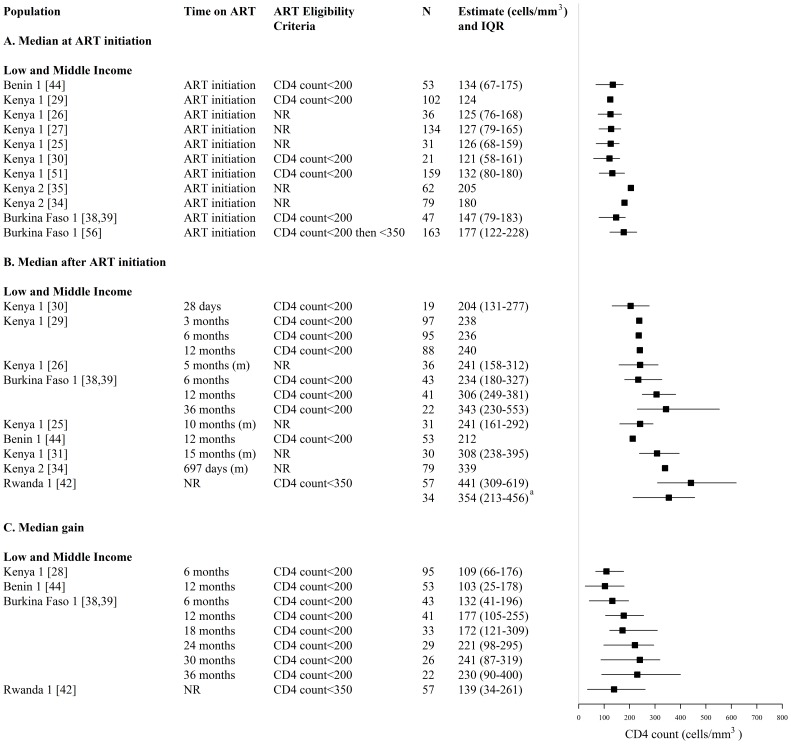
Forest plot of (A) median CD4 count and (B) median gains in CD4 count among HIV-infected FSWs on ART and starting ART. Study estimates are grouped by country income and ordered by time on ART. ^a^ N refers to a subset of FSWs who were eligible for ART upon HIV diagnosis and enrolled in HIV care following HIV diagnosis. ART  =  antiretroviral, FSW  =  female sex workers, CI  =  confidence interval, NR  =  not reported, N =  sample size of FSWs available for each outcome, N_p_ =  number of independent study populations, m =  median, IQR  =  interquartile range.

**Figure 10:**
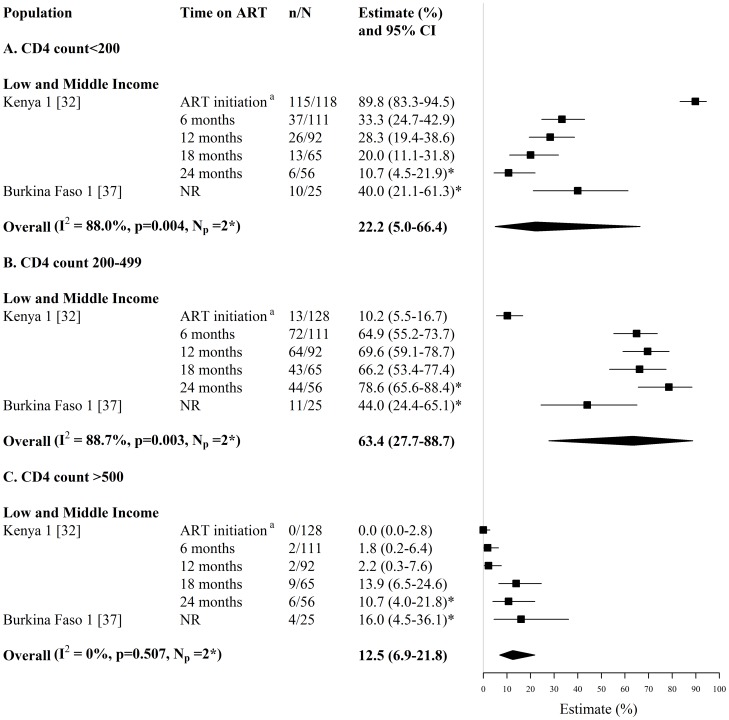
Forest plot of the fraction of HIV-infected FSWs on ART and starting ART with (A) CD4 counts <200 cells/mm^3^ (B) CD4 counts 200–499 cells/mm^3^ and (C) CD4 counts >500 cells/mm^3^. Study estimates are grouped by country income and ordered by time on ART. The star symbol (*) highlights the study estimates (one per study population) included in the pooled overall estimates. For studies providing estimates over multiple time-periods, only one estimate was used for pooling (the most recent estimate from that study). I^2^ and p-values are the measures of heterogeneity used. ^a^ ART initiation criteria is CD4 count <200 cells/mm^3^. ART  =  antiretroviral, FSW  =  female sex workers, CI  =  confidence interval, NR  =  not reported, n =  number of FSWs with each outcome, N =  sample size of FSWs available for each outcome, N_p_ =  number of independent study populations.

Information on CD4 counts at and/or after ART initiation (either median CD4 count, median CD4 count gain, or fraction with CD4 counts >500, 200–499 or <200 cells/mm^3^), were available from 17 studies reporting on 5 independent study populations in Africa [25]–[32], [34], [35], [37]–[39], [42], [44], [51], [56] (Figures 9 &10, Table S5). Across the studies, the CD4 count criteria for ART initiation was either ≤200 cells/mm^3^ or not reported, with one study reporting a change in ART initiation criteria from CD4 count <200 cells/mm^3^ to CD4 count <350 cells/mm^3^ (Table S5).

Median CD4 counts at and/or after ART initiation were provided by 14 studies reporting on 5 independent study populations in Benin, Kenya, Burkina Faso, and Rwanda (N_s_ = 14, N_p_ = 5) (Figure 9A & 9B, Table S5A). Across 4 of these study populations, the median CD4 count ranged between 121 and 205 cells/mm^3^ at ART initiation (N_s_ = 12, N_p_ = 4), and 204 to 343 cells/mm^3^ after 1 to 36 months on ART (N_s_ = 9, N_p_ = 4) (Figure 9A & 9B, Table S5A).

Median gains in CD4 count following ART initiation, were provided by 5 studies reporting on 4 independent study populations in Benin, Kenya, Burkina Faso, and Rwanda (N_s_ = 5, N_p_ = 4) (Figure 9C, Table S5A). Across 3 of these study populations, median gains in CD4 counts after 6 to 36 months on ART, ranged between 103 and 241 cells/mm^3^ (N_s_ = 4, N_p_ = 3) (Figure 9C, Table S5A).

Estimates of the fraction of FSWs with CD4 counts <200, 200–499 or >500 cells/mm^3^ at and/or after ART initiation were provided by 2 studies reporting on 2 independent study populations in Kenya and Burkina Faso (N_s_ = 2, N_p_ = 2) (Figure 10, Table S5B). In the Kenyan study, the fraction of FSWs with CD4 counts ≤200 cells/mm^3^, decreased from 90% at ART initiation to 11% after 24 months on ART [32] (Figure 10A, Table S5B). When pooling the most recent study estimates from the 2 study populations, the overall pooled fractions of FSWs on ART with CD4 counts <200 cells/mm^3^, 200–499 cells/mm^3^, and >500 cells/mm^3^, were 22% (95% CI: 5%–66%, I^2^ = 88%, N_s_ = N_p_ = 2), 63% (95% CI: 28%–89%, I^2^ = 89%, N_s_ = N_p_ = 2), and 13% (95% CI: 7%–22%, I^2^ = 0%, N_s_ = N_p_ = 2), respectively [32], [37] (Figure 10).

## Discussion

Despite the recent momentum to scale-up ART coverage worldwide, there is a concerning lack of published data on HIV treatment among FSWs [61]. We found only thirty-nine studies, from fourteen different countries in Africa, Asia, North America, South America, and Central America and the Caribbean that reported information on the ART cascade among FSWs (Figure 2). Of these studies, the majority reported estimates of either current ART use or median CD4 counts at and/or after ART initiation, with very few providing estimates of treatment attrition, adherence and viral suppression among FSWs.

ART uptake among HIV-infected FSWs was particularly variable across the different studies and settings. Pooled estimates of ART initiation in HIV-infected FSWs were higher among studies which collected data on HIV-infected FSWs exclusively in the HAART era versus studies which collected data spanning the pre-HAART and HAART eras, and pooled estimates of ever ART use among HIV-infected FSWs were higher in high-income countries compared to low- and middle-income countries. Conversely, pooled estimates of current ART use among HIV-infected FSWs were similar between high-income countries and low- and middle-income countries. However, there was substantial between-study heterogeneity for all pooled estimates of ART uptake. Varied study periods, different follow-up times and different ART eligibility criteria across studies could all contribute to the variability in study estimates. Nevertheless, pooled estimates of current ART use among HIV-infected FSWs are similar to estimates of current ART use among HIV-infected women in Burkina Faso (27%) and among HIV-infected female IDUs in Canada (30%) [62], [63]. Likewise, in the one study in Rwanda that reported ART initiation among ART-eligible FSWs, the proportion of ART-eligible FSWs in HIV care initiating ART was similar to the proportion of ART-eligible individuals in the Rwandan general population initiating ART (71%) [42].

Data from two African FSW research cohorts suggest that treatment discontinuation, loss to follow-up on ART, and mortality on ART among FSWs may be low, at least over the first few years of ART use [25], [28], [29], [38], [39]. However, these studies were conducted in research contexts where there is likely to be intensive follow-up of participants, and thus better retention on treatment. Even so, total treatment attrition after one year on ART in one of the African FSW cohorts was similar to that found in the sub-Saharan African general population after 12 months on ART (23%) [64]. In addition estimates of loss to follow-up and death on ART among FSWs are similar to if not better than estimates of loss to follow-up and death among women on ART enrolled in cohort studies in South Africa and Tanzania, where 17.5% and 25.6% were loss to follow-up, respectively, and 4.1% and 12.8% died on ART, respectively [65], [66]. Only three studies in Dominican Republic, Canada and the USA reported on the fraction of ART-experienced FSWs that were no longer on ART, and there was substantial between-study heterogeneity. Although the proportion of FSWs in the Dominican Republic that reported ever but not current ART use was similar to that reported by women from the general population in the USA (9%) [67], the two cross-sectional studies in North America found much higher proportions of FSWs self-reporting that they were previously but no longer on ART [24], [48]. However, the sample size of FSWs in these two North American studies was very small.

Adherence among FSWs, in the few studies where it was measured, was found to be consistently high despite different methods of assessment, recall periods and adherence thresholds. The pooled estimate of adherence among FSWs is similar to that reported for the general population in sub-Saharan Africa, where 77% were estimated to be ≥80% adherent [68], and is also similar to if not better than that reported for women in the general population, where 62% were estimated to be ≥90% adherent [69]. Adherence counselling in 4 studies [23], [38], [39], [44], monthly support groups in one study [60], and directly administered therapy in another study [30], could have contributed to the high adherence seen in these FSW study populations.

Limited data also indicate that overall just over half of FSWs initiating ART are achieving viral suppression, which is much lower than the pooled estimate of ART adherence seen among FSWs. However, there was considerable between-study heterogeneity in the pooled estimate of viral suppression, and the varied limits of viral load detection used to define viral suppression, which ranged from <50 copies/ml to <300 copies/ml, may have influenced the results. The cohort of FSWs in Burkina Faso which used the highest limit of viral load detection to define viral suppression (<300 copies/ml), had the highest estimates of viral suppression, and these were comparable to estimates of viral suppression (defined as viral load <400 copies/ml) seen among women from the general population in South Africa, where 87%–93% of women were virally suppressed after 12 to 36 months on ART [70]. The FSWs in Burkina Faso, who had the highest levels of viral suppression, also had high levels of adherence [38], [39]. Thus, the lower estimates of viral suppression could also reflect lower adherence rates among FSWs in those study settings.

For a number of FSWs, improvements in CD4 count were also achieved within 6 to 24 months of ART initiation, and these were comparable to the median gains in CD4 count achieved among women in the general population from South Africa and the UK after 6 to 12 months on ART [70], [71].

To date, this is the first study to systematically review and quantify the ART cascade among FSWs globally, which builds on a limited review already undertaken by the authors [15]. Available data indicate that FSWs can achieve promising levels of ART uptake, adherence, retention, and treatment response, at least in the short-term and in these research settings, which in some cases may even be comparable to that seen in the general population over similar time-periods. However, there are a number of limitations to this systematic review. In particular, the review and meta-analysis were limited by the scarcity and heterogeneity of eligible studies. The included studies, the majority of which had small sample sizes of HIV-infected FSWs, reported data on only twenty-one different FSW study populations from only fourteen countries in Asia, Africa, North America, South America, and Central America and the Caribbean, which limits the generalizability of these findings to FSWs elsewhere (Figure 2). Most of the included studies were also research cohorts, thus these results are of limited generalizability to FSWs in other routine programme (non-research) settings. FSWs enrolling in research cohorts may be more motivated to adhere to and remain on treatment, and there are likely to be a number of other HIV prevention and support services available to FSWs in these cohorts, which coupled with intense follow-up and intensive adherence counselling, could help to improve ART uptake, retention, adherence and treatment outcomes among FSWs in these studies [72], [73]. Our pooled analyses are also limited by the heterogeneity between studies, but still provide useful overall estimates of engagement in HIV treatment among FSWs. Study periods were particularly variable across studies, and this is likely to influence HIV treatment outcomes, in particular ART uptake. The time periods of data collection spanned 24 years across the different study settings, and in some studies collection of data on HIV-infected FSWs started in the pre-HAART era. To address this issue, whenever possible we conducted sub-group analyses for ART uptake outcomes by time-period of data collection (i.e. data collected in the pre-HAART to HAART era versus HAART era alone). We found that this had an effect on pooled estimates of ART initiation, and although substantial between-study heterogeneity remained, this could indicate that it might not be appropriate to aggregate all data on ART uptake regardless of study period. In the majority of cohort studies, follow-up time on ART was also relatively short, so long term attrition and treatment outcomes in FSWs remain unknown. Lack of reporting on local ART eligibility criteria also made it more difficult to interpret estimates of ART uptake. In addition, adherence to medication was most frequently measured by either self-reports or pill counts, both of which are prone to biases, such as social desirability and recall bias (Table S6). Different types of recruitment settings and sampling methods may have also introduced selection bias into the included studies. However, it was difficult to fully assess risk of selection bias as very few studies reported sampling methodology (Table S6). Some studies reported using random samples, whilst a few others reported the use of non-probability sampling methods, such as snowball sampling and convenience sampling. Snowball sampling and convenience sampling are commonly used when studying hard-to-reach populations, but are likely to have introduced bias into the selection of participants [74]. A couple of studies also reported the use of respondent-driven sampling, which is a type of snowball sampling that aims to reduce bias by taking into account the non-random patterns of participant recruitment [74]. The different recruitment settings and strategies also highlight the diversity of FSW samples across studies (Table S6). Pooled estimates may mask the potential differences in HIV treatment and care across the varied contexts and settings of sex work. It is also possible that women who do not self-identify as FSWs may have been missed at study recruitment, so in some cases outcome data is likely to be restricted to self-identifying FSWs. Thus, results may not be generalizable to indirect FSWs, who may not have been captured in these samples. Publication bias may have also affected our summary estimates, but due to the small numbers of studies that reported each outcome we could not explore this in a meaningful way. In addition, as we did not search for available data in the grey literature (our review focussed on peer-reviewed published literature), we have likely excluded some ART data that may have been available, for example, from reports of integrated biological and behavioural assessment (IBBA) surveys among FSWs.

Despite the limitations, the results from this review are encouraging, and show that even though FSWs are considered a hard population to reach for HIV prevention and care, research cohorts in particular have achieved success at enrolling and retaining FSWs on ART with good adherence and treatment response. Importantly, this review also has important public health implications since it highlights large gaps in the spatial distribution of ART cascade estimates among FSWs (Figure 2) and the paucity of data on the ART cascade among FSWs in routine (non-research) programme settings. These data gaps are concerning, and impede our understanding of ‘real-life’ HIV treatment and care among FSWs and potential feasibility and effectiveness of targeting FSWs for expanded ART prevention strategies. It is crucial that routine programme data on HIV care and treatment among FSWs is collected and disseminated, in order to better characterise ART cascade outcomes among FSWs (see Box S1 for a summary of our recommendations for future data collection). In particular, information on the percentage of all HIV-infected FSWs receiving ART, and how this evolves over time is needed. Estimates of ART use are likely to be affected by ART initiation criteria, HIV testing policies and testing uptake, as well as rates of linkage to and retention in HIV care prior to ART initiation. So in order to interpret data on ART uptake among FSWs, information on the evolution of ART eligibility criteria over time, the CD4 count at which FSWs are diagnosed and started treatment, as well as the percentage of HIV-infected FSWs who receive HIV testing and are linked to HIV care is required. Furthermore, gathering additional short and long-term data on treatment retention, adherence and viral suppression among FSWs, using standardised indicators is crucial. In 2010, the WHO recommended a number of different early warning indicators to use for monitoring of HIV drug resistance in national ART programmes, which included the following ART care indicators: % of patients receiving ART lost to follow-up at 12 month, % of patients retained on first-line ART at 12 months, % of patients with 100% on-time drug pickups during the first 12 months of ART or during a specified time period, % patient adherence to antiretroviral therapy by pill count or other standardized measure, and % of patients with viral load <1000 copies/mm^3^ at 12 months [75]. Gathering and reporting data on these types of ART care indicators for sex workers in routine surveys of sex workers, existing sex worker programmes, and new research studies of HIV care and treatment among FSWs, could help to better characterise the ART cascade among FSWs in different settings and contexts.

When scaling up ART programmes for FSWs, it will also be important to elucidate and overcome the barriers that FSWs face to initiating ART, remaining on treatment and adhering to ART. Many individual-level and structural-level barriers, such as stigma, discrimination, violence, and drug-use, often prevent FSWs from accessing HIV services and practicing HIV prevention and harm reduction, and these barriers may also influence the ability of FSWs to traverse through the HIV care cascade [76]–[79]. In two of the studies included in this systematic review, FSWs reported that inability to attend regular medical appointments, potential loss of wages from visiting ART centres, prior negative experiences with the health care system, the stigmatizing attitude of medical staff, and fear of adverse consequences as a result of others knowing their HIV status and occupation, were some of the factors that prevented them from initiating ART [24], [46]. In three of the studies included in this systematic review, FSWs also reported that running out of pills, not being at home to take pills, feelings of sickness when taking pills, and potential loss of clients if seen taking ART during work hours, were factors that prevented them from adhering to treatment [42], [44], [46]. Another qualitative study among FSWs in Zimbabwe also found that discrimination from medical staff, shame, and anxiety about being known to be a sex worker, reduced the ability of FSWs to attend HIV treatment services [80]. Travelling times to health clinics and additional financial costs were also reported as barriers to treatment [80]. It is essential that new or ongoing HIV intervention and ART programmes among FSWs try to address the structural and social barriers to HIV treatment that FSWs' face. For example, *Abriendo Puertas*, is an ongoing integrated HIV care and prevention intervention for FSWs in Dominican Republic, that aims to promote HIV care and preventative behaviours among FSWs [53]. This intervention has four key components, including individual counselling and education, peer HIV service navigation and support, clinical care health provider sensitisation training, and community solidarity and mobilisation, which together aim to provide a multi-level response to the social and structural factors that prevent FSWs engaging and remaining in care and adhering to treatment [53]. In Zimbabwe, the Sisters with a Voice (SWV) programme, which provides free STI/HIV services and assisted referrals to ART centers for FSWs, has also initiated numerous measures to try and reduce the stigma, discrimination and barriers that FSWs face at public health clinics, including training and sensitisation for nurses at public ART centers and community mobilisation among FSWs [80].

In conclusion, existing studies in a limited number of research settings indicate that it is it is feasible to enrol and retain FSWs on ART with good adherence, at least in the short-term and in these research contexts. However, there is an evident gap in global data on ART cascade outcomes among FSWs. More data, particularly long-term data, from a larger number of FSW study populations and non-research settings, needs to be collected and disseminated. When scaling-up ART programmes for FSWs, the barriers to HIV care and treatment that they face also need to be addressed.

## Supporting Information

Table S1 **ART uptake outcomes.**
(DOCX)

Table S2 **Treatment attrition outcomes.**
(DOCX)

Table S3 **Adherence to ART.**
(DOCX)

Table S4 **Viral Suppression.**
(DOCX)

Table S5 **CD4 Counts at and/or after ART Initiation.**
(DOCX)

Table S6 **Risk of Bias Assessment.**
(DOCX)

Box S1 **Recommendations for Future Data Collection.**
(DOCX)

Checklist S1 **PRISMA Checklist.**
(DOCX)
