# Supplementary material for: Antiretroviral Therapy Uptake, Attrition, Adherence and Outcomes among HIV-Infected Female Sex Workers: A Systematic Review and Meta-Analysis
Source: PLoS One. 2014 Sep 29;9(9):e105645. doi: 10.1371/journal.pone.0105645 (PMC4179256; doi:10.1371/journal.pone.0105645)
Supplement: Table S1 — ART uptake outcomes. (DOCX) [file pone.0105645.s001.docx]

**Table S1: ART uptake outcomes**

| **Population Code** | **Study Reference** | **Outcome** | **Period (year if known)** | **Estimate (%) (95%CI)** | **n/N** | **CD4 count criteria for ART initiation ^f^** |
| --- | --- | --- | --- | --- | --- | --- |
| **A. ART uptake among all HIV-infected FSWs** | | | | | | |
| Brazil 1 | Schuelter-Trevisol *et al,*  2007 [[4](#_ENREF_48)7] | Current ART Use | Enrolment (2003 - 2004) | 25.0 (0.6 - 80.6) | 1/4 | Not reported |
| Burkina Faso 1 | Low *et al,*  2014 [[5](#_ENREF_58)7] | Current ART Use | Enrolment (2007 - 2011) | 69.8 (63.8 - 75.3) * | 180/258 | Not reported |
|  | Low *et al,*  2011 [37] | Current ART Use | Enrolment (2003 - 2005) | 9.5 (6.3 - 13.6) | 26/273 | ≤ 200 cells/mm^3^ |
|  | Konate *et al,* 2011 [38] | Initiated ART | Follow-up (2003 - 2005) | 27.8 (21.2 - 35.2) * | 47/169 | < 200 cells/mm^3^ |
| Canada 1 | Reddon *et al,* 2011 [[4](#_ENREF_47)5] ^a^ | Current ART Use | Enrolment (2005) | 39.8 (29.5 - 50.8)* | 35/88 | Not reported |
| Canada 2 | Cox *et al*, 2014 [[5](#_ENREF_59)8] ^a^ | Current ART Use | Enrolment (2003 - 2013) | 72.1 (56.3 - 84.7)* | 31/43 | Not reported |
| Canada 3 | Shannon *et al,* 2005 [24] | Current ART Use | Enrolment (2003) | 41.2 (24.7 - 59.3) * | 14/34 | Not reported |
|  |  | Ever Used ART | Enrolment (2003) | 67.6 (49.5 - 82.6) * | 23/34 | Not reported |
| Dominican Republic 1 | Donastorg *et al*, 2014 [[5](#_ENREF_54)3] | Current ART use | Enrolment (2012 - 2013) | 72.4 (66.6 - 77.7) * | 194/268 | Not reported |
|  |  | Ever Used ART | Enrolment (2012 - 2013) | 78.4 (72.9 - 83.1) * | 210/268 | Not reported |
| El Salvador 1 | Dennis *et al*, 2013 [[5](#_ENREF_53)2] | Current ART use | Enrolment (2008) | 0.0 (0.0 - 9.0) * | 0/39 | Not reported |
| India 1 | Chakrapani *et al,* 2009 [46] | Current ART Use | Enrolment (2007) | 21.1 (6.1 - 45.6) * | 4/19 | < 200 cells/mm^3^ |
| India 2 | Jadhav *et al*, 2013 [[5](#_ENREF_56)5] | Current ART use | Enrolment (2011) | 41.8 (37.8 - 45.8) * | 252/603 | Not reported |
| India 3 | Becker *et al*, 2012 [[4](#_ENREF_42)0] ^a^ | Current ART Use | At death (2008 - 2009) | 48.6 (31.4 - 66.0) * | 17/35 | Not reported |
| Kenya 1 | Balkus *et al,* 2013 [[49](#_ENREF_50)] | Current ART use | At T.*vaginalis* diagnosis (1993 - 2010) | 23.0 (18.3 - 28.4)* | 65/282 | Not reported |
|  | Graham *et al,* 2013 [5[4](#_ENREF_55)] | Initiated ART | Follow-up (1993 - 2009): |  |  |  |
|  |  |  | 4-24 months post-infection | 0.4 (0.0 - 2.4) | 1/232 | < 200 cells/mm^3^ |
|  |  |  | 24 months post-infection | 6.3 (3.1 - 11.3) | 10/159 | < 200 cells/mm^3^ |
|  | McClelland *et al,* 2011 [[3](#_ENREF_36)3] | Initiated ART | Follow-up (1993 - 2006) | 18.9 (15.8 - 22.4) | 108/571 | Not reported |
|  | McClelland *et al,* 2010 [[32](#_ENREF_35)] | Initiated ART | Follow-up (1993 - 2008) | 20.4 (17.9 - 23.1) * | 197/966 | < 200 cells/mm^3^ |
| Kenya 2 | McKinnon *et al,* 2010 [[3](#_ENREF_37)4] ^a^ | Current ART Use | Enrolment (2009) | 29.2 (25.6 - 33.0) * | 177/607 | Not reported |
|  | Lester *et al,*  2009 [[3](#_ENREF_39)6] | Current ART Use | Enrolment (Unknown date) | 33.3 (21.4 - 47.1) | 19/57 | Not reported |
| Kenya 3 | Graham *et al*, 2013 [[6](#_ENREF_61)0] ^a^ | Ever Used ART | Enrolment (2005 - 2011) | 13.9 (8.0 - 21.9)* | 15/108 | ≤ 250 cells/mm^3^ |
| Rwanda 1 | Braunstein *et al,*  2011 [[4](#_ENREF_44)2] | Initiated ART | Follow-up (12-36 months after HIV diagnosis in 2008/2009) | 47.5 (38.3 - 56.8) * | 57/120 ^b^ | < 350 cells/mm^3^ |
| Russia 1 | Tyurina *et al*, 2013 [59] ^a^ | Current ART Use | Enrolment (2007 - 2011) | 7.1 (1.5 - 19.5) * | 3/42 | Not reported |
| Thailand 1 | Kilmarx *et al,* 2000 [[2](#_ENREF_26)2] | Initiated ART | Follow-up (1991 - 1998) | 1.6 (0.3 - 4.5) * | 3/194 | Not reported |
| USA 1 | Comulada *et al,* 2003 [[4](#_ENREF_49)8] ^a^ | Current ART use | Enrolment (1999 - 2000) | 0.0 (0.0 - 84.2) | 0/2 ^c^ | Any CD4 count |
|  |  |  | Enrolment (1999 - 2000) | 47.6 (25.7 - 70.2) * | 10/21 ^c^ | Any CD4 count |
|  |  | Ever Used ART | Enrolment (1999 - 2000) | 50.0 (1.3 - 98.7) | 1/2 ^c^ | Any CD4 count |
|  |  |  | Enrolment (1999 - 2000) | 90.5 (69.6 - 98.8) * | 19/21 ^d^ | Any CD4 count |
| USA 2 | Kalokhe *et al,* 2012 [[4](#_ENREF_43)1] ^a^ | Current ART Use | Enrolment (2006 - 2010) | 17.4 (7.8 - 31.4) * | 8/46 | Not reported |
| Vietnam 1 | Dean *et al,*  2011 [4[3](#_ENREF_45)] ^a^ | Ever Used ART | Enrolment (2008 - 2009) | 22.1 (15.4 - 30.2) * | 29/131 | Not reported |
| Zimbabwe 1 | Cowan *et al,* 2013 [[5](#_ENREF_51)0] | Current ART use | Enrolment (2009) | 31.3 (27.1 - 35.6) * | 150/480 | Not reported |
| **B. ART uptake among ART-eligible HIV-infected FSWs** | | | | | | |
| Rwanda 1 | Braunstein *et al,* 2011 [[4](#_ENREF_44)2] | Initiated ART | Follow-up (12-36 months after HIV diagnosis in 2008/2009) | 87.2 (72.6 - 95.7) | 34/39 ^e^ | < 350 cells/mm^3^ |

FSW – female sex worker, ART – antiretroviral therapy, HIV – human immunodeficiency virus, WISH – Women’s Information and Safe House.
^a^ Data was provided by study authors.
^b^ N = HIV-infected FSWs who were enrolled in care after HIV diagnosis.
^c^ Active FSWs.
^d^ Active and Former FSWs.
^e^ N = HIV-infected FSWs ART-eligible at baseline who were enrolled in care after HIV diagnosis.
^f^ ART initiation criteria is not related to the denominator (N) for part A of the table.
* highlights the study estimates used in pooled estimates.
