# Supplementary material for: Antiretroviral Therapy Uptake, Attrition, Adherence and Outcomes among HIV-Infected Female Sex Workers: A Systematic Review and Meta-Analysis
Source: PLoS One. 2014 Sep 29;9(9):e105645. doi: 10.1371/journal.pone.0105645 (PMC4179256; doi:10.1371/journal.pone.0105645)
Supplement: Table S2 — Treatment attrition outcomes. (DOCX) [file pone.0105645.s002.docx]

**Table S2: Treatment attrition outcomes**

| **Population Code** | **Study Reference** | **Outcome** | **Period (year if known)** | **Estimate (%) (95%CI)** | **n/N** |
| --- | --- | --- | --- | --- | --- |
| Burkina Faso 1 | Huet *et al,* 2011 [39] &  Konate *et al,* 2011 [38] | Cumulative mortality (any cause) | First 12 months on ART | 6.4 (1.3 - 17.5) ^c^ * | 3/47 |
|  |  |  | First 36 months on ART | 8.5 (2.4 - 20.4) ^c^ | 4/47 |
|  |  | Cumulative LFU | Median of 32 months on ART | 4.3 (0.5 - 14.5) * | 2/47 |
| Canada 3 | Shannon *et al,* 2005 [[24](#_ENREF_17)] | No longer on ART | Enrolment (2003) | 39.1 (19.7 - 61.5) * | 9/23 |
| Dominican Republic 1 | Donastorg *et al*, 2014 [[53](#_ENREF_54)] | No longer on ART | Enrolment (2012 - 2013) | 7.6 (4.4 - 12.1) * | 16/210 |
| Kenya 1 | Day *et al,* 2013 [[5](#_ENREF_52)1] | Cumulative mortality (any cause) | Median of 41 months on ART | 4.4 (1.8 - 8.9) | 7/159 |
|  | Graham *et al,* 2012 [29] & Graham *et al* 2010 [28] | Cumulative mortality (any cause) | First 3 months on ART ^b^ | 2.0 (0.2 - 6.9) | 2/102 |
|  |  |  | First 6 months on ART | 2.0 (0.2 - 6.9) | 2/102 |
|  |  |  | First 12 months on ART ^b^ | 4.9 (1.6 - 11.1) * | 5/102 |
|  |  | Cumulative LFU | First 3 months on ART ^b^ | 2.9 (0.6 - 8.4) | 3/102 |
|  |  |  | First 6 months on ART | 3.9 (0.1 - 9.7) | 4/102 |
|  |  |  | First 12 months on ART ^b^ | 5.9 (2.2 - 12.4) * | 6/102 |
|  | Graham *et al,* 2010 [28] | Discontinued ART | First 6 months on ART | 3.9 (1.1 - 9.7) | 4/102 |
|  | Graham *et al,* 2012 [29] | Discontinued ART | First 12 months on ART | 9.8 (4.8 - 17.3) | 10/102 |
|  | Masese *et al,* 2011 [[2](#_ENREF_28)5] | Cumulative LFU | Median of 10 months on ART | 4.9 (0.6 - 16.5) | 2/41 |
| Kenya 3 | Graham *et al*, 2013 [[6](#_ENREF_61)0] ^a^ | Cumulative mortality (any cause) | Time on ART not reported | 0.0 (0.0 - 30.9)* | 0/10 |
|  |  | Cumulative LFU | Time on ART not reported | 10.0 (0.3 - 44.5)* | 1/10 |
| USA 1 | Comulada *et al,* 2003 [[4](#_ENREF_49)8] | No longer on ART | Enrolment (1999 - 2000) | 47.4 (24.5 - 71.1) * | 9/19 |

FSW – female sex worker, ART – antiretroviral therapy, LFU – loss to follow-up, WISH – Women’s Information and Safe House, pyrs – person years.
^a^ Data was provided by study authors.
^b^ Outcome estimate only reported in Graham *et al,* 2012 [29].
^c^ 95% confidence interval reported in study.
* highlights the study estimates used in pooled estimates.
