# Supplementary material for: Antiretroviral Therapy Uptake, Attrition, Adherence and Outcomes among HIV-Infected Female Sex Workers: A Systematic Review and Meta-Analysis
Source: PLoS One. 2014 Sep 29;9(9):e105645. doi: 10.1371/journal.pone.0105645 (PMC4179256; doi:10.1371/journal.pone.0105645)
Supplement: Table S3 — Adherence to ART. (DOCX) [file pone.0105645.s003.docx]

**Table S3: Adherence to ART**

| **Population Code** | **Study Reference** | **Threshold; Method of Assessment and Recall Period** | **Period (year if known)** | **Estimate (%) (95%CI)** | **n/N** | **Adherence Support** |
| --- | --- | --- | --- | --- | --- | --- |
| Burkina Faso 1 | Huet *et al,* 2011 [39] & Konate *et al,* 2011 [38] | ≥ 95 adherence; assessed by pill count (no. of pills taken/no. prescribed) in the past 30 days | 6 months after ART initiation ^b^ | 83.3 (67.2 - 93.6) ^c^ | Not known | Adherence counselling |
|  |  |  | 12 months after ART initiation ^b^ | 92.1 (78.6 - 98.3) ^c^ | Not known | Adherence counselling |
|  |  |  | 36 months after ART initiation | 100.0 (54.1 -100.0) ^c^ | Not known | Adherence counselling |
| Canada 3 | Deering *et al*, 2009  [[2](#_ENREF_27)3] | 100% adherence; assessed by self-report (no missed pills) in the past week | First 13 weekly meetings attended of PDI | 87.2 | Not known | Adherence counselling |
|  |  |  | Last 13 weekly meetings attended of PDI | 90.4 | Not known | Adherence counselling |
| Dominican Republic 1 | Donastorg *et al*, 2014 [[5](#_ENREF_54)3] | 100% adherence; assessed by self-report (no missed pills) in past 4 days | Median of 5 years since HIV diagnosis | 73.8 (67.3 - 79.6) * | 155/210 | Not reported |
| Kenya 1 | Masese *et al,* 2011 [[2](#_ENREF_28)5] | ≥ 95% adherence; assessed by pill count (no. of pills taken/no. prescribed) in the past month | Median of 10.3 months on ART | 67.7 (48.6 - 83.3%) * | 21/31 | Not reported |
|  | Graham *et al*, 2007 [[3](#_ENREF_33)0] ^a^ | 100% adherence; assessed by pill count (no. of pills taken/no. prescribed) at each study visit in the past month | First 28 days on ART | 90.5 (69.6 - 98.8) | 19/21 | Directly administered therapy used at each visit |
| Kenya 3 | Graham *et al*, 2013 [6[0](#_ENREF_61)] ^a^ | ≥ 95 adherence; assessed by visual analog scale (percent of pills reported missed) in the past 30 days | Time on ART not reported | 80.0 (44.4 - 97.5) * | 8/10 | Monthly support groups to promote adherence |
| Rwanda 1 | Braunstein *et al,* 2011 [4[2](#_ENREF_44)] | 100% adherence; assessed by self-report (no missed pills) since ART initiation | 12-36 months post-HIV diagnosis | 66.7 (52.9 - 78.6) | 38/57 | Not reported |
|  |  | 100% adherence; assessed by self-report (no missed pills) in the past 3 days | 12-36 months post-HIV diagnosis | 86.0 (74.2 - 93.7) * | 49/57 | Not reported |
| USA 1 | Comulada *et al,* 2003 [[4](#_ENREF_49)8] ^a^ | ≥ 90% adherence; assessed by self-report (no. of pills taken/no. of pills prescribed) in the past 3 days | Enrolment | 66.7 (29.9 - 92.5) | 6/9 | Not reported |

FSW – female sex worker, ART – antiretroviral therapy, PDI – peer-driven intervention
^a^ Data was provided by study authors
^b^ Outcome estimate only reported in Huet *et al,* 2011 [[39](#_ENREF_19)]
^c^ 95% confidence interval reported in study
* highlights the study estimates used in pooled estimates.
