# Supplementary material for: Antiretroviral Therapy Uptake, Attrition, Adherence and Outcomes among HIV-Infected Female Sex Workers: A Systematic Review and Meta-Analysis
Source: PLoS One. 2014 Sep 29;9(9):e105645. doi: 10.1371/journal.pone.0105645 (PMC4179256; doi:10.1371/journal.pone.0105645)
Supplement: Table S4 — Viral Suppression. (DOCX) [file pone.0105645.s004.docx]

**Table S4: Viral Suppression**

| **Population Code** | **Study Reference** | **Outcome** | **Period (year if known)** | **Estimate (%) (95%CI)** | **n/N** |
| --- | --- | --- | --- | --- | --- |
| Burkina Faso 1 | Low *et al*, 2014 [[5](#_ENREF_57)6] | Undetectable PVL (≤300 copies/mm^3^) | Median of 3.4 years on ART | 48.3 (40.7 - 56.0) * | 84/174 |
|  | Huet *et al,* 2011 [[39](#_ENREF_19)] &  Konate *et al,* 2011 [[3](#_ENREF_41)8] | Undetectable PVL (≤300 copies/mm^3^) | 6 months after ART initiation | 79.4 (62.1 - 91.3) ^c^ | 27/34 |
|  |  |  | 12 months after ART initiation | 77.1 (59.8 - 89.6) ^c^ | 27/35 |
|  |  |  | 18 months after ART initiation | 80.0 (61.4 - 92.3) ^c^ | 24/30 |
|  |  |  | 24 months after ART initiation ^b^ | 87.5 (47.4 - 99.7) ^c^ | 7/8 |
|  |  |  | 30 months after ART initiation ^b^ | 75.0 (42.8 - 94.5) ^c^ | 9/12 |
|  |  |  | 36 months after ART initiation | 81.8 (59.7 - 94.8) ^c^ | 18/22 |
|  |  | Primary virological success rate (defined as 2 samples ≤300 copies/mm^3^) | Total follow-up on ART (median 32 months) | 76.7 (61.4 - 88.2) ^c^ | 36/47 |
| Dominican Republic 1 | Donastorg *et al*, 2014 [[5](#_ENREF_54)3] ^a^ | Undetectable PVL (<50 copies/mm^3^) | Median of 5 years since HIV diagnosis | 59.6 (52.3 - 66.6) * | 115/193 |
| Kenya 1 | Graham *et al,* 2010 [28] | Undetectable PVL (≤100 copies/mm^3^) | 3 months after ART initiation | 40.2 (30.4 - 50.7) | 39/97 |
|  |  |  | 6 months after ART initiation | 72.6 (62.5 - 81.3) * | 69/95 |
| Vietnam 1 | Dean *et al,* 2011 [[4](#_ENREF_45)3] ^a^ | Undetectable PVL  (≤180 copies/mm^3^) | Enrolment (2008 – 2009) | 44.8 (26.5 - 64.3) * | 13/29 |

FSW – female sex worker, ART – antiretroviral therapy, PVL – plasma viral load
^a^ Data was provided by study authors.
 ^b^ Outcome estimate only reported in Huet *et al,* 2011 [[39](#_ENREF_19)].
^c^ 95% confidence interval reported in study.
* highlights the study estimates used in pooled estimates.
