# Supplementary material for: Antiretroviral Therapy Uptake, Attrition, Adherence and Outcomes among HIV-Infected Female Sex Workers: A Systematic Review and Meta-Analysis
Source: PLoS One. 2014 Sep 29;9(9):e105645. doi: 10.1371/journal.pone.0105645 (PMC4179256; doi:10.1371/journal.pone.0105645)
Supplement: Table S5 — CD4 Counts at and/or after ART Initiation. (DOCX) [file pone.0105645.s005.docx]

**Table S5: CD4 Counts at and/or after ART Initiation**

| **Population Code** | **Study Reference** | **Outcome** | **Period (year if known)** | **Estimate** | **N or n/N** |
| --- | --- | --- | --- | --- | --- |
| **A. CD4 count (cells/mm^3^)** | | | | | |
| Benin 1 | Diabaté *et al*, 2011 [[44](#_ENREF_46)] | Median CD4 count | ART initiation ^b^ | 134 (IQR: 67 -175) | 53 |
|  |  |  | 12 months after ART initiation | 212 | 53 |
|  |  | Median CD4 count gain | 12 months after ART initiation | 103 (IQR: 25 - 178) | 53 |
| Burkina Faso 1 | Low *et al,* 2014 [[5](#_ENREF_57)6] | Median CD4 count | ART initiation ^c^ | 177 (IQR: 122 - 228) | 163 |
|  | Huet *et al,* 2011 [[39](#_ENREF_19)] &  Konate *et al,* 2011 [[38](#_ENREF_41)] | Median CD4 count | ART initiation ^b d^ | 147 (IQR: 79 - 183) | 47 |
|  |  |  | 6 months after ART initiation | 234 (IQR: 180 - 327) | 43 |
|  |  |  | 12 months after ART initiation | 306 (IQR: 249 - 381) | 41 |
|  |  |  | 36 months after ART initiation | 343 (IQR: 230 - 553) | 22 |
|  |  | Median CD4 count gain | 6 months after ART initiation | 132 (IQR: 41 - 196) | 43 |
|  |  |  | 12 months after ART initiation | 177 (IQR: 105 - 255 | 41 |
|  |  |  | 18 months after ART initiation ^d^ | 172 (IQR: 121 - 309) | 33 |
|  |  |  | 24 months after ART initiation ^d^ | 221 (IQR: 98 - 295) | 29 |
|  |  |  | 30 months after ART initiation ^d^ | 240 (IQR: 87 - 319) | 26 |
|  |  |  | 36 months after ART initiation | 230 (IQR: 90 - 400) | 22 |
| Kenya 1 | Day *et al,* 2013 [[5](#_ENREF_52)1] | Median CD4 count | ART initiation ^b^ | 132 (IQR: 80 -180) | 159 |
|  | Graham *et al,* 2012 [29] | Median CD4 count | ART initiation ^b^ | 124 (range: 2 - 443) | 102 |
|  |  |  | 3 months after ART initiation | 238 (range: 65 - 631) | 97 |
|  |  |  | 6 months after ART initiation | 236 (range: 65 - 692) | 95 |
|  |  |  | 12 months after ART initiation | 240 (range: 93 - 875) | 88 |
|  | Graham *et al*, 2011 [[2](#_ENREF_29)6] | Median CD4 count | ART initiation ^e^ | 125 (IQR: 76 - 168) | 36 |
|  |  |  | Median of 5 months on ART | 241 (IQR: 158 - 312) | 36 |
|  | Masese *et al,* 2011 [[2](#_ENREF_28)5] | Median CD4 count | ART initiation ^e^ | 126 (IQR: 68 - 159) | 31 |
|  |  |  | Median of 10.3 months on ART | 241 (IQR: 161 - 292) | 31 |
|  | Gitau *et al*, 2010 [31] | Median CD4 count | Median of 15 months on ART | 308 (IQR: 238 - 395) | 30 |
|  | Graham *et al* 2010 [[28](#_ENREF_31)] | Median CD4 count gain | 6 months after ART initiation | 109 (IQR: 66 - 176) | 95 |
|  | Graham *et al*, 2009 [27] | Median CD4 count | ART initiation  ^e^ | 127 (IQR: 79 - 165) | 134 |
|  | Graham *et al*, 2007 [[3](#_ENREF_33)0] | Median CD4 count | ART initiation ^b^ | 121 (IQR: 58 - 161) | 21 |
|  |  |  | 28 days after ART initiation | 204 (IQR: 131 - 277) | 19 |
| Kenya 2 | Mawji *et al*, 2012 [[3](#_ENREF_38)5] | Median CD4 count | ART initiation ^e^ | 205 (range: 70 -1028) | 62 |
|  | McKinnon *et al*, 2010 [[3](#_ENREF_37)4] | Median CD4 count | ART initiation ^e^ | 180 (range: 3-851) | 79 |
|  |  |  | Median of 697 days on ART | 339 | 79 |
| Rwanda 1 | Braunstein *et al,* 2011 [[4](#_ENREF_44)2] | Median CD4 count | 12-36 months post-HIV diagnosis | 441 (IQR: 309 - 612) | 57 |
|  |  |  |  | 354 (IQR: 213 - 456) | 34 ^f^ |
|  |  | Median CD4 count gain | 12-36 months post-HIV diagnosis | 139 (IQR: 34 - 261) | 57 |
| **B. Stratified CD4 count (cells/mm^3^)** | | | | | |
| Burkina Faso 1 | Low *et al*, 2011 [37] | Fraction with CD4 count  < 200 | Enrolment (time on ART not known) | 40.0% (95%CI: 21.1% - 61.3%) * | 10/25 |
|  |  | Fraction with CD4 count 200 - 499 | Enrolment (time on ART not known) | 44.0% (95%CI: 24.4% - 65.1%) * | 11/25 |
|  |  | Fraction with CD4 count  > 500 | Enrolment (time on ART not known) | 16.0% (95%CI: 4.5% - 36.1%) * | 4/25 |
| Kenya 1 | McClelland *et al,* 2010 [[3](#_ENREF_35)2] ^a^ | Fraction with CD4 count  < 200 | ART initiation ^b^ | 89.8% (95%CI: 83.3% - 94.5%) | 115/128 |
|  |  |  | 6 months after ART initiation | 33.3% (95%CI: 24.7% - 42.9%) | 37/111 |
|  |  |  | 12 months after ART initiation | 28.3% (95%CI: 19.4% - 38.6%) | 26/92 |
|  |  |  | 18 months after ART initiation | 20.0% (95%CI: 11.1% - 31.8%) | 13/65 |
|  |  |  | 24 months after ART initiation | 10.7% (95%CI: 4.5% - 21.9%) * | 6/56 |
|  |  | Fraction with CD4 count  200 - 499 | ART initiation ^b^ | 10.2% (95%CI: 5.5% - 16.7%) | 13/128 |
|  |  |  | 6 months after ART initiation | 64.9% (95%CI: 55.2% - 73.7%) | 72/111 |
|  |  |  | 12 months after ART initiation | 69.6% (95%CI: 59.1% - 78.7%) | 64/92 |
|  |  |  | 18 months after ART initiation | 66.2% (95%CI: 53.4% - 77.4%) | 43/65 |
|  |  |  | 24 months after ART initiation | 78.6% (95%CI: 65.6% - 88.4%) * | 44/56 |
|  |  | Fraction with CD4 count  ≥ 500 | ART initiation ^b^ | 0.0% (95%CI: 0.0% - 2.8%) | 0/128 |
|  |  |  | 6 months after ART initiation | 1.8% (95%CI: 0.2% - 6.4%) | 2/111 |
|  |  |  | 12 months after ART initiation | 2.2% (95%CI: 0.3% - 7.6%) | 2/92 |
|  |  |  | 18 months after ART initiation | 13.9% (95%CI: 6.5% - 24.6%) | 9/65 |
|  |  |  | 24 months after ART initiation | 10.7% (95%CI: 4.0% - 21.8%) * | 6/56 |

FSW – female sex worker, ART – antiretroviral therapy
^a^ Data was provided by study authors
^b^ ART eligibility criteria ≤ 200 cells/mm^3
c^ ART eligibility criteria ≤ 200 cells/mm^3^ prior to 2009 and ≤ 350 cells/mm^3^ from 2009
^d^ Outcome estimate only reported in Huet *et al,* 2011 [[39](#_ENREF_19)]
^e^ ART eligibility criteria not reported
^f^ Subset of individuals who were eligible for ART upon HIV diagnosis and enrolled in HIV care following HIV diagnosis
* highlights the study estimates used in pooled estimates
