# Supplementary material for: Antiretroviral Therapy Uptake, Attrition, Adherence and Outcomes among HIV-Infected Female Sex Workers: A Systematic Review and Meta-Analysis
Source: PLoS One. 2014 Sep 29;9(9):e105645. doi: 10.1371/journal.pone.0105645 (PMC4179256; doi:10.1371/journal.pone.0105645)
Supplement: Table S6 — Risk of Bias Assessment. (DOCX) [file pone.0105645.s006.docx]

**Table S6: Risk of Bias Assessment**

| **Population Code** | **Study [Reference]** | **Study Design** | **Setting/Recruitment** | **Sampling** | **Outcome Measurement Methods** |
| --- | --- | --- | --- | --- | --- |
| Benin 1 | Diabaté *et al*,  2011 [[4](#_ENREF_46)4] | PC | FSWs were recruited from a FSW dedicated medical center in Cotonou. | Consecutive | Adherence assessed by self-report of missed doses during the four days before each visit. CD4 count measured using Flow Cytometry |
| Brazil 1 | Schuelter-Trevisol *et al*, 2007 [[4](#_ENREF_48)7] | CS | FSWs in the port City of Imbituba registered with an NGO were recruited. | Not specified | Not specified |
| Burkina  Faso 1 | Low *et al*, 2014 [[5](#_ENREF_57)6] | PC | See Huet *et al*. 2011 | See Huet *et al*. 2011 | Viral load quantified using real-time PCR assay. CD4 count measured using FACScan. |
|  | Low *et al*, 2013 [[5](#_ENREF_58)7] | PC | See Huet *et al*. 2011 | See as Huet *et al*. 2011 | Not specified |
|  | Huet *et al*, 2011 [[39](#_ENREF_19)] | PC | FSWs from Burkina Faso were recruited through a network of peer educators | Not specified | Attrition measured by study team during follow-up. Adherence assessed by monthly pill count. CD4 count measured using FACScan. Viral load quantified using real-time PCR assay. |
|  | Konate *et al*, 2011 [38] | PDI | See Huet *et al*. 2011 | See Huet *et al*. 2011 | See Huet *et al*, 2011. Other outcome measurement methods not specified. |
|  | Low *et al*, 2011 [[37](#_ENREF_40)] | PC | See Huet *et al*. 2011 | See Huet *et al*. 2011 | Not specified |
| Canada 1 | Reddon *et al*, 2011 [[4](#_ENREF_47)5] | PC | FSWs were recruited into ACCESS study from Vancouver’s Downtown Eastside (drug use and HIV epicenter). | Snowball sampling (with extensive street outreach) | Confidential linkage of ART use data from province wide centralised ART dispensation program. |
| Canada 2 | Cox *et al*, 2014 [[58](#_ENREF_59)] | PC | HIV-HCV co-infected individuals were recruited from 18 centres across Canada. | All eligible individuals invited to participate. | Not specified |
| Canada 3 | Deering *et al*,  2009 [[2](#_ENREF_27)3] | PDI | FSWs were recruited into PDI through referral by HIV specialist, family care physician or other health provider, friend or by self-referral. | Not specified | 100% adherence in past week self-reported. |
|  | Shannon *et al*,  2005 [24] | CS | FSWs were recruited at a FSW Drop-In Centre Society in Vancouver during evening drop-in hours. | Random sampling. | Current and prior ART use self-reported. |
| Dominican Republic 1 | Donastorg *et al*, 2014 [[5](#_ENREF_54)3] | CS | Peer navigators approached FSWs who they knew were living with HIV. Only FSWs reporting HIV positive status were invited to participate. | Non-random hybrid sampling approach | ART use, treatment attrition and adherence self-reported. |
| El Salvador 1 | Dennis *et al*, 2013 [[5](#_ENREF_53)2] | CS | FSWs were recruited in San Salvador and San Miguel by their peers. | Respondent-driven sampling | ART use self-reported. |
| India 1 | Chakrapani *et al*, 2009 [46] | CS | FSWs were recruited through NGOs that provide services to FSWs in Chennai. | Purposive sampling | ART use self-reported. |
| India 2 | Jadhav *et al*, 2013 [[5](#_ENREF_56)5] | CS | FSWs who disclosed their HIV status to peer educators following HIV testing in each district (Bagalkot, Belgaum & Bijapur) were recruited. | Stratified random sampling. | Not specified |
| India 3 | Becker *et al*, 2012 [[4](#_ENREF_42)0] | PC | FSWs were recruited from different villages in 3 districts in Karnataka. | Not specified | ART use verbally reported by informants of deceased FSWs. |
| Kenya 1 | Balkus *et al*, 2013 [49] | PC | See Graham *et al*, 2007 | See Graham *et al*, 2007 | Not specified |
|  | Day *et al*, 2013 [[5](#_ENREF_52)1] | PC | See Graham *et al*, 2007 | See Graham *et al*, 2007 | Assessment of CD4 count not specified – likely to be Cytosphere or FACS count (Masese *et al*, 2012). |
|  | Graham *et al*, 2013 [[5](#_ENREF_55)4] | PC | See Graham *et al*, 2007 | See as Graham *et al*, 2007 | Not specified |
|  | Graham *et al*,  2012 [[2](#_ENREF_32)9] | PC | See Graham *et al*, 2007 | See Graham *et al*, 2007 | CD4 count measured using FACS count. Other outcome measurement methods not specified. |
|  | Graham *et al*, 2011 [[2](#_ENREF_29)6] | PC | See Graham *et al*, 2007 | See Graham *et al*, 2007 | Assessment of CD4 count not specified – likely to be Cytosphere or FACS count (see Masese *et al*, 2012). |
|  | Masese *et al*, 2011[[2](#_ENREF_28)5] | PC | FSWs were recruited from larger research cohort of FSWs in Mombasa. | Not specified | Adherence assessed by monthly pill count. CD4 count measured using Cytosphere up until Oct 2004 and FACS count thereafter. Other outcome measurement methods not specified. |
|  | McClelland *et al*, 2011 [[3](#_ENREF_36)3] | CS | See Graham *et al*, 2007 | See Graham *et al*, 2007 | Not specified |
|  | Gitau *et al*, 2010 [[3](#_ENREF_34)1] | PC | See Graham *et al*, 2007 | See Graham *et al*, 2007 | CD4 count measured using FACS count. |
|  | Graham *et al*, 2010 [28] | PC | See Graham *et al*, 2007 | Same as Graham *et al*, 2007 | Viral load quantification using the Gen-Probe HIV-1 viral load assay. CD4 count measured using FACS count. Other outcome measurement methods not specified. |
|  | McClelland *et al*, 2010 [[3](#_ENREF_35)2] | PC | See Graham *et al*, 2007 | See Graham *et al*, 2007 | CD4 count measured using Cytosphere up until Oct 2004 and FACS count thereafter. Other outcome measurement methods not specified. |
|  | Graham *et al*, 2009 [27] | PC | See Graham *et al*, 2007 | See Graham *et al*, 2007 | Assessment of CD4 count not specified – likely to be Cytosphere or FACS count (see Masese *et al*, 2011). |
|  | Graham *et al*, 2007 [[3](#_ENREF_33)0] | PC | FSWs were recruited from the municipal STD clinic in Mombasa. | Not specified | Adherence assessed by pill count at each study visit: day 1, 2, 4, 7, 14 & 28. CD4 count measured using Cytosphere. |
| Kenya 2 | Mawji *et al*, 2012 [[3](#_ENREF_38)5] | RCC | FSWs were recruited from an established cohort of FSWs from Nairobi. | Not specified | Not specified |
|  | McKinnon *et al*, 2010 [[3](#_ENREF_37)4] | PC | See Mawji *et al.* 2012 | See Mawji *et al.*2012 | Program data on ART use. Method of measuring CD4 count not reported. |
|  | Lester *et al*, 2009 [[3](#_ENREF_39)6] | CS | FSWs were recruited from FSW cohort in Nairobi during scheduled outpatient research visits. | Not specified | Not specified |
| Kenya 3 | Graham *et al*,  2013 [61] | PC | FSWs residing in the clinic catchment area were recruited either at venues where high-risk sex was common or by trained peer mobilizers via personal networks | Not specified | Adherence monitored at each refill by a 30-day visual analog scale. Other outcome measurement methods not specified. |
| Russia 1 | Tyurina *et al*, 2013 [[6](#_ENREF_60)1] | PC | Not specified | Not specified | ART use information obtained from medical record review. |
| Rwanda 1 | Braunstein *et al*, 2011 [[4](#_ENREF_44)2] | PC | FSWs were recruited via community meetings in 3 districts in Kigali. | Not specified | ART use self-reported. Adherence since ART initiation, or within past 3 days at study visit, was self-reported. CD4 cytometry used. |
| Thailand 1 | Kilmarx *et al*, 2000 [[2](#_ENREF_26)2] | PC | FSWs in Chiang Rai were approached by study staff in the provincial STD clinic, other medical clinics in the province, and at their workplaces. | Not specified | ART use self-reported. |
| USA 1 | Comulada *et al*, 2003 [[4](#_ENREF_49)8] | CS | Participants were recruited from HIV/AIDS clinical care sites, social services agencies, street outreach programs, advertisements and community announcements. | Convenience sampling | Current and prior ART use self-reported. Adherence assessed by self-report during past 3 days. |
| USA 2 | Kalokhe *et al*,  2012 [[4](#_ENREF_43)1] | CS | FSWs were recruited from hospital inpatient services in Miami and Atlanta. | Not specified | ART use self-reported |
| Vietnam 1 | Dean *et al*, 2011 [[4](#_ENREF_45)3] | CS | FSWs were recruited from 5 centers across Vietnam. | Not specified | ART use self-reported. Validated viral load RT-PCR assay. |
| Zimbabwe 1 | Cowan et al, 2013 [[5](#_ENREF_51)0] | CS | FSWs were recruited from three study sites (Mutare, Hwange and Victoria Falls). | Respondent-driven sampling | ART use self-reported |

FSW – female sex worker, ART – antiretroviral therapy, ACCESS – AIDS Care Cohort to evaluate Exposure to Survival Services, STD – sexually transmitted diseases, NGO – non-governmental organisation, PC – prospective cohort, CS – cross-sectional, RCT – randomised controlled trial, RCC – retrospective case-control, PDI – peer driven intervention
