# Supplementary material for: Antiretroviral Therapy Uptake, Attrition, Adherence and Outcomes among HIV-Infected Female Sex Workers: A Systematic Review and Meta-Analysis
Source: PLoS One. 2014 Sep 29;9(9):e105645. doi: 10.1371/journal.pone.0105645 (PMC4179256; doi:10.1371/journal.pone.0105645)
Supplement: Box S1 — Recommendations for Future Data Collection. (DOCX) [file pone.0105645.s007.docx]

**Box S1: Recommendations for Future Data Collection**

| - Routine programme data on HIV care and treatment among FSWs needs to be collected and disseminated - Representative data on the % of FSWs with HIV receiving ART (and how this evolves over time) is particularly critical - To interpret and help improve ART uptake among FSWs, information on the following data in FSWs is also needed:   - ART eligibility criteria (and how this evolved over time)   - CD4 counts at diagnosis and treatment initiation   - Rate of HIV testing of FSWs and linkage to HIV care of those diagnosed with HIV - Standardised indicators and additional short and long-term data of treatment retention, adherence and viral suppression among FSWs, are also needed - Ideally, standard indicators of ART coverage and care for FSWs would be integrated into routine surveys of sex workers, existing sex worker programmes, and new research studies of HIV treatment and care among FSWs |
| --- |
